# Increased colon cancer risk after severe *Salmonella* infection

**DOI:** 10.1371/journal.pone.0189721

**Published:** 2018-01-17

**Authors:** Lapo Mughini-Gras, Michael Schaapveld, Jolanda Kramers, Sofie Mooij, E. Andra Neefjes-Borst, Wilfrid van Pelt, Jacques Neefjes

**Affiliations:** 1 National Institute for Public Health and the Environment (RIVM), Antonie van Leeuwenhoeklaan 9, Bilthoven, the Netherlands; 2 Department of Infectious Diseases and Immunology, Utrecht University, Yalelaan 1, Utrecht, the Netherlands; 3 Division of Epidemiology, the Netherlands Cancer Institute (NKI), Plesmanlaan 121, Amsterdam, the Netherlands; 4 Department of Pathology, Free University Medical Center (VUmc), Boelelaan 1117, Amsterdam, the Netherlands; 5 Department of Chemical Immunology, Leiden University Medical Center (LUMC), Einthovenweg 20, Leiden, the Netherlands; Kliniken der Stadt Köln gGmbH, GERMANY

## Abstract

**Background:**

Colon cancer constitutes one of the most frequent malignancies. Previous studies showed that *Salmonella* manipulates host cell signaling pathways and that *Salmonella* Typhimurium infection facilitates colon cancer development in genetically predisposed mice. This epidemiological study examined whether severe *Salmonella* infection, usually acquired from contaminated food, is associated with increased colon cancer risk in humans.

**Methods and findings:**

We performed a nationwide registry-based study to assess colon cancer risk after diagnosed *Salmonella* infection. National infectious disease surveillance records (1999–2015) for Dutch residents aged ≥20 years when diagnosed with salmonellosis (*n* = 14,264) were linked to the Netherlands Cancer Registry. *Salmonella*-infected patients were laboratory-confirmed under medical consultation after 1–2 weeks of illness. These datasets also contained information on *Salmonella* serovar and type of infection. Colon cancer risk (overall and per colon subsite) among patients with a diagnosed *Salmonella* infection was compared with expected colon cancer risk in the general population. Data from the nationwide registry of histo- and cytopathology (PALGA) and Statistics Netherlands (CBS) allowed assessing potential effects of age, gender, latency, socioeconomic status, genetic predisposition, inflammatory bowel disease (IBD), and tumor features. We found that compared to the general population, colon cancer risk was significantly increased (standardized incidence ratio [SIR] 1.54; 95%CI 1.09–2.10) among patients with *Salmonella* infection diagnosed <60 years of age. Such increased risk concerned specifically the ascending/transverse colon (SIR 2.12; 95%CI 1.38–3.09) after *S*. Enteritidis infection (SIR 2.97; 95%CI 1.73–4.76). Salmonellosis occurred more frequently among colon cancer patients with pre-infectious IBD, a known risk factor for colon cancer. Colon tumors of patients with a history of *Salmonella* infection were mostly of low grade.

**Conclusions:**

Patients diagnosed with severe salmonellosis have an increased risk of developing cancer in the ascending/transverse parts of the colon. This risk concerns particularly *S*. Enteritidis infection, suggesting a contribution of this major foodborne pathogen to colon cancer development.

## Introduction

It is estimated that over 20% of the global cancer burden is attributable to infectious agents [1]. In contrast to virally induced cancers [2–4], bacteria have been largely neglected as factors contributing to cancer [5], with only a few bacterial infections linked to cancer development to date [6, 7]. This is best established for *Helicobacter pylori* in connection with gastric cancer [8] and Mucosa-Associated Lymphoid Tissue (MALT) lymphoma [9], and for *Salmonella* Typhi and gallbladder carcinoma in chronic typhoid carriers [10–13].

Bacteria may contribute to cancer development through inflammation, induction of DNA damage by toxins, metabolites, and/or manipulation of host cell signaling pathways during their infection cycle [14, 15]. For instance, *H*. *pylori* has been shown to support gastric cell transformation by secreting toxin CagA that activates the c-Met receptor and induces signaling [16], with chronic inflammation acting as a contributing factor. Bacteria can also alter the cell biology of the host during the infection cycle, as illustrated by *Salmonella* species that manipulate host cell signaling pathways to enforce bacterial uptake, intracellular survival and egress [17]. *Salmonella* secretes effector proteins into host cells that activate the host AKT and ERK pathways. These pathways are also activated in many cancers, and are essential for transforming pretransformed cells [13]. Another *Salmonella* effector AvrA activates host β-catenin signaling and also promotes colon carcinogenesis in mice [18, 19]. If by virtue of altered host cell signaling bacteria provide one step towards cancer development [20], it is conceivable that bacterial infections would then also increase cancer risk. This would be expected particularly under conditions of long-lasting infections, where the chance of targeting an already pre-transformed cell is higher. This has been illustrated for *Salmonella* Typhimurium infection in normal and genetically predisposed (APC+/-) mice, with the latter developing colon carcinoma [13]. Notwithstanding these findings and the demonstrated cellular localization of AvrA in inflamed, colorectal tumors and their precursor lesions in both experimental mouse models and human clinical specimens [19], it remains unclear whether the many *Salmonella* infections occurring in the human population constitute a risk factor for colon cancer.

Roughly 93.8 million people are infected with *Salmonella* species annually [21]. While over 2,500 different serovars of *Salmonella enterica* subspecies *enterica* exist, serovars Typhimurium and Enteritidis are responsible for around 70% of salmonellosis cases in Europe. As for many enteric infections, most salmonellosis cases are not reported, as they generally present with mild and self-limiting symptoms requiring no medical attention. Consequently, it is estimated that for every salmonellosis case reported to the national infectious disease surveillance systems in Europe, approximately 57 cases go unreported [22].

With 694,000 deaths in 2012, colon cancer is a major cause of cancer morbidity and mortality worldwide [23], especially for older patients, as colon cancer incidence increases markedly after the age of 60 years [8, 24]. In 2014, 10,319 residents in the Netherlands were diagnosed with colon cancer and 3,682 residents died due to colon cancer [25]. Factors predisposing for colon cancer include Inflammatory Bowel Disease (IBD) [26, 27] and genetic mutations [28, 29]. Colon cancer incidence increases over the years as a function of largely unknown risk factors [30, 31]. To address whether *Salmonella* infections constitute yet another risk factor for colon cancer, we compared the incidence of colon cancer among Dutch residents with a reported history of *Salmonella* infection to that in the general Dutch population. Moreover, we examined potential effects of gender, age, latency, socioeconomic status (SES), type of infection, IBD, genetic predisposition and tumor pathological features on the association between *Salmonella* infection and colon cancer.

## Methods

### Ethics statement

The researchers obtained written permission to use and link the different data sets after anonymization. The contract numbers for the different data sets are: NCR (K16.147 and K15.257) and PALGA (lzv2016-72).

### Data collection

We performed a retrospective cohort study based on three linked registries described in detail elsewhere [32–34]. The first registry is maintained by the Dutch National Institute for Public Health and the Environment (RIVM) and contains national surveillance data on reported human salmonellosis cases from the Netherlands’ laboratory surveillance network, which includes 16 regional public health laboratories covering 64% of the general Dutch population [32, 33]. At the time of analysis, this database included a total of 28,117 unique records of patients with a laboratory-confirmed *Salmonella* spp. infection in the Netherlands diagnosed between January 1999 and December 2015 with associated metadata, i.e. birth date, gender, date of diagnosis, residence location, isolated *Salmonella* serovar, and type of infection (enteric, septicemic, other) (S1 Table). The second database, maintained by the Dutch Association of Comprehensive Cancer Centers (IKNL), derives from the Netherlands Cancer Registry (NCR). This registry covers all Dutch residents, the data are more than 95% complete, and includes 140,685 patients diagnosed with colon cancer (ICD-O-3 codes: C180-C189) in 1999–2015. These data also include the colon subsite (ascending, transverse, and descending/sigmoid) in which the tumor has been diagnosed. The third database (Dutch Nationwide Network and Registry of Histo- and Cytopathology, PALGA) contains the pathology records of all patients in the Netherlands and is a countrywide database since 1991 [35].

### Data anonymization and linkage

Statistics Netherlands (CBS) acted as a trusted third party for data anonymization and linkage by adding a Record Identification Number (RIN) as unique identifier for each individual in all databases. Birth date, gender, residence location, and date of registration formed the basis for the derivation of the RIN numbers. To this end, CBS used a reference database containing all mutations due to death or relocation in the Dutch population since 1995, including a complete housing history of all Dutch residents. After the RIN numbers were added, all personal identifiers were removed. Based on RIN numbers, patients with a reported *Salmonella* infection in the RIVM database were linked to the NCR data on patients with diagnosed colon cancer. The RIN numbers also allowed coupling to other CBS information, such as the date of death and the standardized household income (or “spendable income”, adjusted for the size and composition of the household) used as a proxy for SES. The linkage with the PALGA data first required de-anonymization by CBS and then transfer of the data to IKNL before transfer to PALGA for record classification. To this end, the colon cancer patient group with a reported *Salmonella* infection (*n* = 65) was mixed with a three-fold larger randomly selected age- and gender-matched group of colon cancer patients without such infection history (*n* = 194). Their pathology records were then provided to the pathologist for further classification, without information on infection. The pathologist then reported the classifications to CBS for anonymization and addition of RIN numbers before releasing the data for analysis.

### Participants and classification

**Fig 1:**
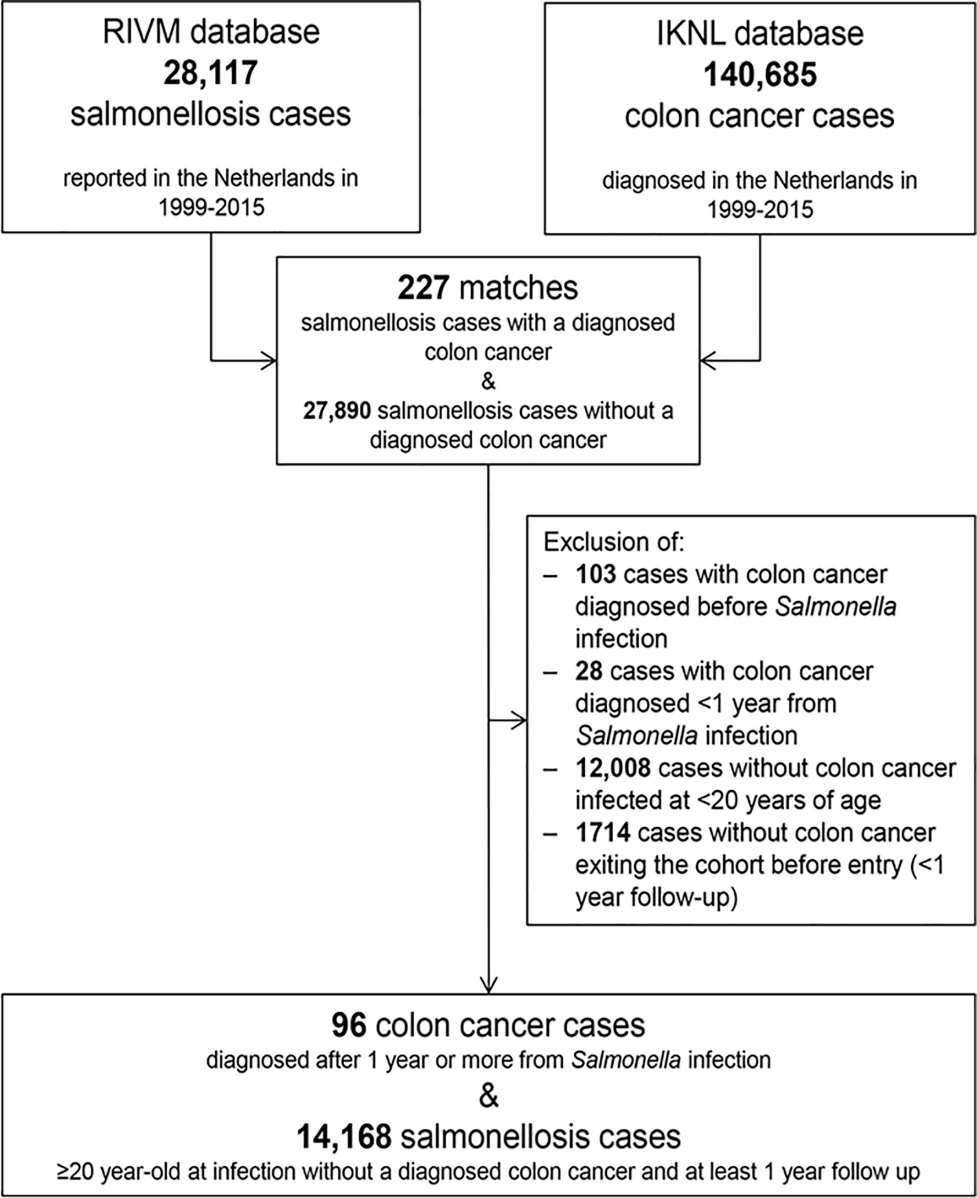
Schematic representation of the data management process. Number of patients in the two linked databases, as well as the number of patients excluded from further analyses according to each inclusion criterion.

All data sets were cleared from duplicates. Patients with the reporting date of *Salmonella* infection falling before the start (January 1^st^, 1999) or after the end (December 31^st^, 2015) of the study period were censored. After linking the records of the 28,117 salmonellosis cases in the RIVM database to those of the 140,685 colon cancer patients in the NCR database, 227 matches (i.e. salmonellosis cases with a diagnosed colon cancer) were found, whereas 27,890 salmonellosis cases did not have a diagnosed colon cancer. In the main analysis, the follow-up period expressed in years at risk started one year after the date of *Salmonella* infection and ended at the date of colon cancer diagnosis, date of death, or end of the study period, whichever came first. We excluded 103 salmonellosis cases diagnosed with colon cancer before *Salmonella* infection and 28 salmonellosis cases having colon cancer diagnosed <1 year from *Salmonella* infection. Patients younger than 20 years at *Salmonella* infection (*n* = 12,008) were also excluded as their risk of colon cancer is virtually zero (no colon cancer cases were observed in this age group after matching with *Salmonella* data). Finally, all 1,714 salmonellosis cases without a diagnosed colon cancer that could be followed for less than one year after infection were removed. For patients with multiple *Salmonella* infections reported over time, only the first one was considered. Our final cohort therefore comprised 14,264 salmonellosis cases aged ≥20 years when diagnosed with *Salmonella* infection between 1999 and 2015, and with at least one year follow-up post-infection. Of these, 96 were subsequently diagnosed with colon cancer ≥1 year after the reported *Salmonella* infection (Fig 1).

As colon cancer incidence increases steeply after 60 years of age due to several factors [8, 24] (S1 Fig) that may in principle dilute the hypothesized effect of *Salmonella* infection, we also performed additional analyses considering only those patients younger than 60 years at infection. For sensitivity analysis of the follow-up period, additional analyses were performed with the time at risk starting at 4, 7 or 10 years after infection. As regard to the final PALGA data set for analysis, this comprised 65 colon cancer patients with a reported *Salmonella* infection and 194 age- and gender-matched colon cancer patients without a reported history of *Salmonella* infection to be used as control group.

### Statistical analysis

In the main analysis, time at risk started one year after diagnosis of salmonellosis and ended at the date of colon cancer diagnosis, date of death or January 1^st^, 2016, whichever occurred first. Colon cancer risk among salmonellosis cases was first estimated as compared with colon cancer risk in the Dutch population (i.e. the baseline reference risk) by calculating standardized incidence ratios (SIRs) of colon cancer (overall and per colon subsite) by dividing the observed number of colon cancer patients among those with a reported *Salmonella* infection by the expected number of colon cancers based on subsite-, age-, gender- and calendar year-matched colon cancer incidence rates in the general Dutch population, as derived from NCR figures. SIRs for colon cancer overall and per colon subsite (ascending, transverse, or descending/sigmoid) were then stratified by gender, age at infection (20–39, 40–49, 50–59, 60–69, ≥70 years), follow-up time in years at risk after infection (1–7 and >7 years, according to median follow-up), diagnosed *Salmonella* serovar (Typhimurium and its monophasic variant with antigenic formula 1,4,[5],12:i:-, Enteritidis, or others), and type of infection (enteric, septicemic, or others like urinary tract or wound infections). To increase statistical power and because the risk of cancer in the ascending and transverse colon was comparable, these two adjacent colon subsites were combined. 95% confidence intervals (95%CIs) for SIRs were calculated assuming a Poisson distribution. Tests for heterogeneity and trends in SIRs were performed using Poisson regression analysis of collapsed person-time data.

Joint Cox proportional hazards regression analysis with attained age as time-scale, accounting for death as competing risk, with entry into the at-risk period one year after the age at *Salmonella* infection, was then used to assess associations of gender, age group at infection, infecting *Salmonella* serovar, type of infection and SES (low *vs*. high, according to median standardized household income) with cancer risk in the ascending/transverse colon and descending/sigmoid colon simultaneously within the cohort. In this analysis, only the patients with reported salmonellosis were included, meaning that no increased or decreased risk for colon cancer after *Salmonella* infection can be shown by the within-cohort analysis, as there is no baseline reference risk (i.e. colon cancer incidence in the general population) to be used for comparison. The purpose of within-cohort comparisons was therefore to assess whether there were also differences in cancer incidence in the different portions of the colon altogether as a function of age, gender, serovar, infection type and SES in the salmonellosis patients alone. Proportional hazard assumptions were verified using graphical and residual-based methods. Associations were expressed as adjusted hazard ratios (HRs) and corresponding 95%CIs.

An additional analysis assessed potential effects of IBD, genetic predisposition (mutations in the Ras/Raf/Mapk pathway), microsatellite instability (MSI), tumor stage (0-I, II, III, IV, based on TNM classification) and differentiation in colon cancer patients who experienced salmonellosis *vs*. those who did not. This analysis was performed using multivariable binomial regression models, which in the group of colon cancer patients for which the PALGA records were assessed (*n* = 259) estimated the risk of having had salmonellosis (binary response) as a function of IBD, genetic predisposition, MSI, tumor stage and differentiation, with correction for the matching variables (age and gender). Associations were expressed as adjusted risk ratios (RRs) and corresponding 95%CIs. Retained in the models were only those factors that were significantly associated with the outcome (i.e. having experienced reported *Salmonella* infection) or that changed the RRs of the other covariates >10% when removed from the model. In all analyses, p-values <0.05 were considered statistically significant. Statistical analysis was performed using STATA 14 (StataCorp LP, College Station, USA).

## Results

### Description of the cohort

Our cohort comprised 14,264 patients (53.6% women and 46.4% men) aged ≥20 years when diagnosed with *Salmonella* infection between 1999 and 2015 and with at least 1 year follow-up post-infection (Fig 1). The median age at *Salmonella* infection was 46 years (interquartile range [IQR], 29–63), with 70.5% being <60 years at infection. Median follow-up was 7 years (IQR, 3–12). In total, 96 patients were diagnosed with colon cancer ≥1 year after the reported *Salmonella* infection (Fig 1), with a median time between salmonellosis and colon cancer diagnoses of 5 years (IQR, 3–9). At 61 and 91 years of age, cumulative incidence of colon cancer among salmonellosis cases was 1.31% (95%CI, 0.784–1.84%) and 4.83% (95%CI, 3.89–5.79%), respectively (S2 Fig). Of the 14,264 *Salmonella* infections included in the cohort, 89% were enteric infections, 5% were septicemic infections, and 6% were of other types.

### Colon cancer risk after *Salmonella* infection as compared to the population

**Fig 2:**
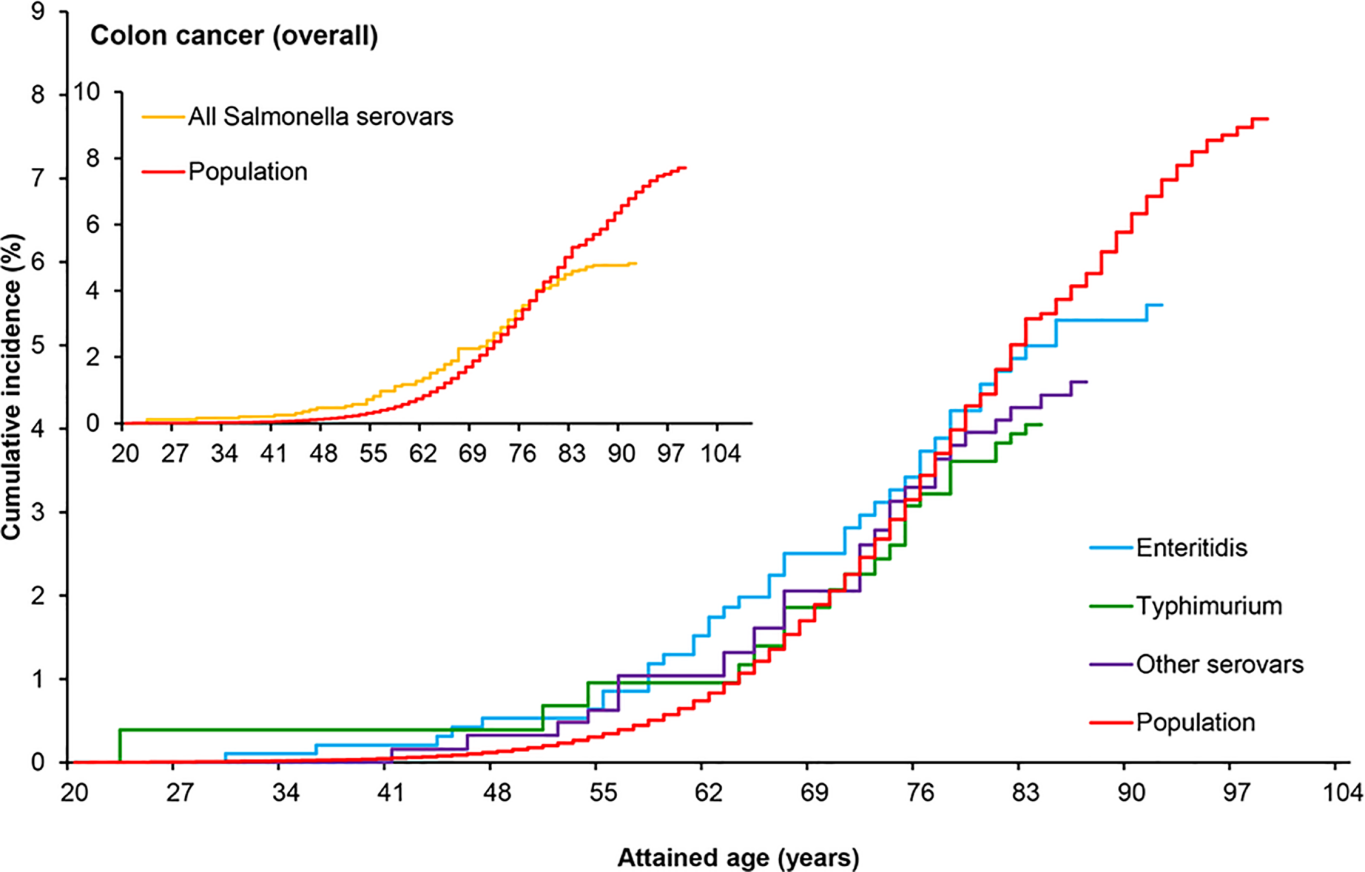
Cumulative incidence of colon cancer in patients with *Salmonella* infection and in the general population. Cumulative incidence of colon cancer over attained age in patients with a reported history of *Salmonella* infection and in the general population. Inset: cumulative incidence of colon cancer in patients with any *Salmonella* serovar infection and in the general population. Main graph: cumulative incidence of colon cancer in patients infected with the two major *Salmonella* serovars (Enteritidis and Typhimurium), the other less often diagnosed *Salmonella* serovars combined, and in the general population.

Colon cancer risk among salmonellosis patients was estimated as compared with the general Dutch population (baseline) by calculating SIRs and corresponding 95%CIs based on colon subsite-, age-, gender- and calendar year-matched colon cancer incidence rates. Compared to the population, cumulative incidence of colon cancer among salmonellosis patients was slightly higher until 70 years of age (Fig 2, inset), but overall colon cancer incidence among salmonellosis patients was not significantly increased (SIR 1.17; 95%CI 0.95–1.43) (Table 1).

**Table 1:** Colon cancer risk by gender and age at *Salmonella* infection.

Gender	Colon cancer (overall)			Ascending & transverse colon			Descending & sigmoid colon		
**All ages ≥20 years**	**Obs**	**Exp**	**SIR (95% CI)**	**Obs§**	**Exp**	**SIR (95% CI)**	**Obs§**	**Exp**	**SIR (95% CI)**
Overall	96	81.9	1.17 (0.95–1.43)	65	44.1	1.48 (1.14–1.88)**	28	32.7	0.86 (0.57–1.24)
Male	47	36.2	1.30 (0.96–1.73)	31	18.3	1.70 (1.15–2.41)**	14	14.3	0.98 (0.54–1.64)
Female	49	45.7	1.07 (0.79–1.42)	34	25.8	1.32 (0.91–1.84)	14	18.4	0.76 (0.42–1.28)
*P-heterogeneity*	*0*.*34*			*0*.*31*			*0*.*50*		
**≥20 and <60 years**									
Overall	39	25.4	1.54 (1.09–2.10)*	26	12.3	2.12 (1.38–3.09)***	11	11	1.00 (0.50–1.79)
Male	21	12.8	1.64 (1.01–2.50)*	14	5.9	2.39 (1.31–4.00)*	6	5.5	1.09 (0.40–2.37)
Female	18	12.5	1.44 (0.85–2.27)	12	6.4	1.87 (0.97–3.26)	5	5.5	0.91 (0.30–2.13)
*P-heterogeneity*	*0*.*68*			*0*.*53*			*0*.*77*		
**Age at infection**	**Obs**	**Exp**	**SIR (95% CI)**	**Obs§**	**Exp**	**SIR (95% CI)**	**Obs§**	**Exp**	**SIR (95% CI)**
20–39 years	7	2.7	2.55 (1.03–5.25)*	3	1.6	1.93 (0.40–5.64)	3	1.3	2.40 (0.50–7.01)
40–49 years	10	6.2	1.62 (0.78–2.98)	7	2.9	2.39 (0.96–4.93)	2	2.9	0.70 (0.08–2.52)
50–59 years	22	16.5	1.34 (0.84–2.02)	16	7.8	2.05 (1.17–3.33)*	6	6.9	0.88 (0.32–1.91)
60–69 years	29	24.2	1.20 (0.80–1.72)	22	12.7	1.74 (1.09–2.63)*	6	9.6	0.62 (0.23–1.36)
≥70 years	28	32.3	0.87 (0.58–1.25)	17	19.1	0.89 (0.52–1.42)	11	12.1	0.91 (0.45–1.63)
*P-heterogeneity*	*0*.*32*			*0*.*89*			*0*.*28*		
*P-trend*	*0*.*01*			*0*.*01*			*0*.*47*		

*p-value <0.05

**p-value <0.01

***p-value <0.001.

§3 colon cancer cases were excluded from the colon subsite-specific analysis as they had cancer involving both the ascending/transverse and descending/sigmoid regions of the colon.

Risk of colon cancer as a whole and per subsite by gender and age at *Salmonella* infection for patients of all ages (≥20 years) and for those <60 years at infection. Observed (Obs) and expected (Exp) numbers of cancers, standardized incidence ratio (SIR) with 95% confidence interval (CI), test of SIR for heterogeneity and trend.

When the different *Salmonella* serovars were considered, cumulative incidence of colon cancer for *S*. Enteritidis-infected patients was higher than in the population (Fig 2). When considering only patients under 60 years of age at the time of *Salmonella* infection, colon cancer risk was significantly increased (SIR 1.54; 95%CI 1.09–2.10) (Table 1), and such risk further increased when considering patients infected with *S*. Enteritidis (SIR 1.86; 1.17–2.82) (Table 2). SIRs did not differ over gender (Table 1).

**Table 2:** Colon cancer risk by follow-up, *Salmonella* serovar and infection type.

**Follow-up time** **(years at risk)**	**Colon cancer (overall)**			**Ascending & transverse colon**			**Descending & sigmoid colon**		
**All ages ≥20 years**	**Obs**	**Exp**	**SIR (95% CI)**	**Obs§**	**Exp**	**SIR (95% CI)**	**Obs§**	**Exp**	**SIR (95% CI)**
1–7 years	55	48.3	1.14 (0.86–1.48)	37	26.1	1.42 (1.00–1.95)	16	19.6	0.82 (0.47–1.33)
>7 years	41	33.6	1.22 (0.88–1.66)	28	17.9	1.56 (1.04–2.26)*	12	13.1	0.92 (0.47–1.60)
*P-heterogeneity*	*0*.*74*			*0*.*69*			*0*.*76*		
**≥20 and <60 years**									
1–7 years	15	10.1	1.49 (0.84–2.46)	11	5.0	2.22 (1.11–3.96)*	3	4.7	0.65 (0.13–1.88)
>7 years	24	15.3	1.57 (1.00–2.33)	15	7.3	2.05 (1.15–3.38)*	8	6.3	1.27 (0.55–2.49)
*P-heterogeneity*	*0*.*88*			*0*.*84*			*0*.*32*		
***Salmonella* serovar**	**Colon cancer (overall)**			**Ascending & transverse colon**			**Descending & sigmoid colon**		
**All ages ≥20 years**	**Obs**	**Exp**	**SIR (95% CI)**	**Obs§**	**Exp**	**SIR (95% CI)**	**Obs§**	**Exp**	**SIR (95% CI)**
Typhimurium	24	21.5	1.12 (0.72–1.66)	13	11.8	1.10 (0.59–1.88)	10	8.4	1.18 (0.57–2.17)
Enteritidis	43	33.2	1.29 (0.94–1.74)	33	17.7	1.86 (1.28–2.61)**	9	13.4	0.67 (0.31–1.27)
Other	29	27.2	1.07 (0.72–1.53)	19	14.5	1.31 (0.79–2.04)	9	10.8	0.83 (0.38–1.58)
*P-heterogeneity*	*0*.*69*			*0*.*21*			*0*.*45*		
**≥20 and <60 years**									
Typhimurium	5	4.8	1.05 (0.34–2.45)	2	2.3	0.86 (0.10–3.10)	3	2.0	1.47 (0.30–4.29)
Enteritidis	22	11.8	1.86 (1.17–2.82)*	17	5.7	2.97 (1.73–4.76)***	4	5.1	0.78 (0.21–2.00)
Other	12	8.8	1.36 (0.71–2.38)	7	4.2	1.65 (0.66–3.40)	4	3.8	1.05 (0.29–2.69)
*P-heterogeneity*	*0*.*43*			*0*.*15*			*0*.*71*		
**Type of infection**	**Colon cancer (overall)**			**Ascending & transverse colon**			**Descending & sigmoid colon**		
**All ages ≥20 years**	**Obs**	**Exp**	**SIR (95% CI)**	**Obs§**	**Exp**	**SIR (95% CI)**	**Obs§**	**Exp**	**SIR (95% CI)**
Enteric	86	71.1	1.20 (0.96–1.48)	59	38.5	1.53 (1.17–1.98)**	24	28.7	0.84 (0.54–1.25)
Septicemic	5	4.1	1.22 (0.40–2.85)	2	2.2	0.90 (0.11–3.24)	3	1.6	1.86 (0.38–5.43)
Other†	5	6.1	0.82 (0.27–1.92)	4	3.4	1.19 (0.32–3.03)	1	2.4	0.42 (0.01–2.33)
*P-heterogeneity*	*0*.*71*			*0*.*68*			*0*.*32*		
**≥20 and <60 years**									
Enteric	38	23.5	1.62 (1.15–2.22)	26	11.4	2.30 (1.49–3.50)***	10	10.1	0.99 (0.47–1.81)
Septicemic	1	0.8	1.30 (0.03–7.22)	0	0.4	0.00 (0.00–10.03)	1	0.3	2.95 (0.08–16.45)
Other†	0	1.1	0.00 (0.00–3.23)	0	0.6	0.00 (0.00–6.70)	0	0.5	0.00 (0.00–7.52)
*P-heterogeneity*	*0*.*98*			*1*.*00*			*0*.*58*		

*p-value <0.05

**p-value <0.01

***p-value <0.001.

§3 colon cancer cases were excluded from the colon subsite-specific analysis as they had cancer involving both the ascending/transverse and descending/sigmoid regions of the colon.

†*Salmonella* isolated from urinary tract or wound infections.

Risk of colon cancer as a whole and per subsite by follow-up time, infecting *Salmonella* serovar and type of infection for patients of all ages (≥20 years) and for those <60 years at infection. Observed (Obs) and expected (Exp) numbers of cancers, standardized incidence ratio (SIR) with 95% confidence interval (CI), test of SIR for heterogeneity.

The colon is morphologically different at subsites, with the ascending and transverse parts being most exposed to *Salmonella* infection, as they are closest to the terminal ileum where such infections mainly reside. For this reason, the analysis was stratified by colon subsite (ascending/transverse or descending/sigmoid colon) showing that salmonellosis patients had significantly increased SIRs of cancer in the ascending/transverse colon, but not in the descending/sigmoid colon, as compared to the general population; this was consistent over gender, age, and follow-up periods (Tables 1 and 2). SIR of cancer in the ascending/transverse colon was 1.48-fold (95%CI 1.14–1.88) increased, and over 2-fold increased (SIR 2.12; 95%CI 1.38–3.09) for patients infected before 60 years of age. Age stratification showed that SIRs of cancer in the ascending/transverse colon among salmonellosis cases decreased to population levels at ≥70 years of age when infected (Table 1). There was no effect of follow-up duration: the SIRs of cancer in the ascending/transverse colon among patients infected before 60 years were 2.22 (95%CI 1.11–3.96) and 2.05 (95%CI 1.15–3.38) at 1–7 and >7 years post-infection, respectively (Table 2).

**Fig 3:**
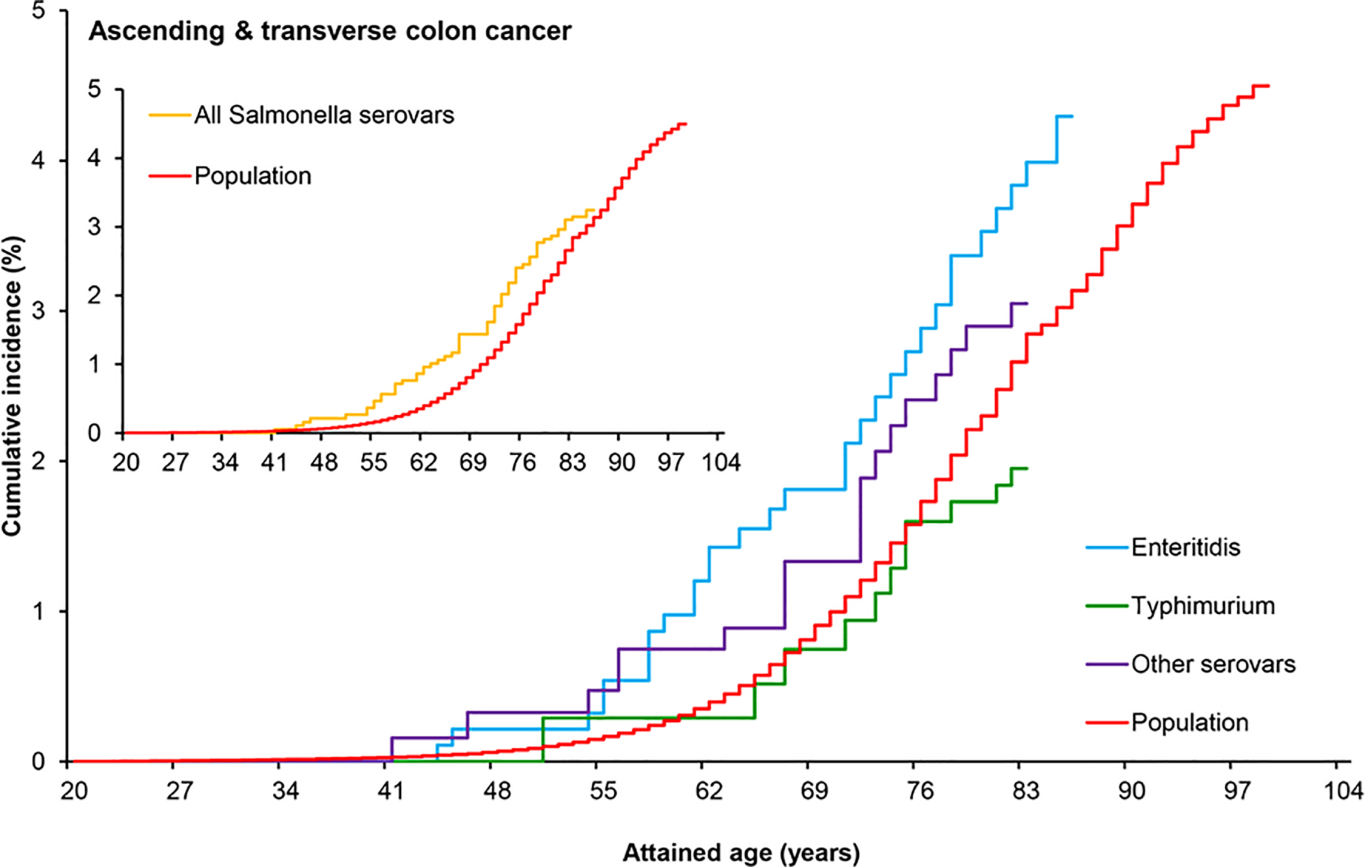
Cumulative incidence of cancer in the ascending and transverse parts of the colon. Cumulative incidence of cancer in the ascending/transverse colon over attained age in patients with a reported history of *Salmonella* infection and in the general population. Inset: cumulative incidence of cancer in the ascending/transverse colon in patients with any *Salmonella* serovar infection and in the general population. Main graph: cumulative incidence of cancer in the ascending/transverse colon in patients infected with the two major *Salmonella* serovars (Enteritidis and Typhimurium), the other less often diagnosed *Salmonella* serovars combined, and in the general population.

The two most common serovars, *S*. Typhimurium and *S*. Enteritidis, differ in epidemiological and biological aspects, which could lead to different risks for colon cancer. The other *Salmonella* serovars were collectively analyzed. Cumulative incidence of colon cancer was highest for patients infected with serovar Enteritidis (Fig 2, main graph), especially when considering the ascending and transverse parts of the colon (Fig 3, main graph) unlike the descending and sigmoid parts of the colon (S3 Fig).

The increased SIR of cancer in the ascending/transverse colon after *S*. Enteritidis infection was as high as 2.97 (95%CI 1.73–4.76) when infection was diagnosed before 60 years of age (Table 2), and ever higher (3.22; 95%CI 1.84–5.23) for patients aged 40–59 years at infection (Table 3). Regarding the type of *Salmonella* infection, significantly increased SIRs were only observed among enteric infections (Tables 2 and 3).

**Table 3:** Colon cancer risk after *Salmonella* Enteritidis infection.

**Follow-up time** **(years at risk)**	**Colon cancer (overall)**			**Ascending & transverse colon**			**Descending & sigmoid colon**		
**All ages ≥20 years**	**Obs**	**Exp**	**SIR (95% CI)**	**Obs§**	**Exp**	**SIR (95% CI)**	**Obs§**	**Exp**	**SIR (95% CI)**
1–7 years	21	17.3	1.22 (0.75–1.86)	15	9.3	1.62 (0.91–2.70)	5	7.20	0.69 (0.23–1.62)
>7 years	22	15.9	1.38 (0.87–2.09)	18	8.5	2.12 (1.30–3.40)**	4	6.23	0.64 (0.18–1.64)
*P-heterogeneity*	*0*.*68*			*0*.*44*			*0*.*91*		
**≥20 and <60 years**									
1–7 years	9	4.3	2.08 (0.95–3.96)	7	2.1	3.30 (1.32–6.76)*	1	2.0	0.50 (0.01–2.77)
>7 years	13	7.5	1.73 (0.92–2.96)	10	3.6	2.80 (1.34–5.13)**	3	3.1	0.97 (0.20–2.82)
*P-heterogeneity*	*0*.*67*			*0*.*74*			*0*.*57*		
**Type of infection**	**Colon cancer (overall)**			**Ascending & transverse colon**			**Descending & sigmoid colon**		
**All ages ≥20 years**	**Obs**	**Exp**	**SIR (95% CI)**	**Obs§**	**Exp**	**SIR (95% CI)**	**Obs§**	**Exp**	**SIR (95% CI)**
Enteric	42	30.2	1.39 (1.00–1.88)*	32	16.1	1.99 (1.36–2.81)***	9	12.3	0.73 (0.34–1.39)
Septicemic	0	1.5	0.00 (0.00.2.40)	0	0.9	0.00 (0.00–4.35)	0	0.6	0.00 (0.00–6.20)
Other†	1	1.5	0.68 (0.02–3.77)	1	0.8	1.24 (0.03–6.92)	0	0.6	0.00 (0.00–6.37)
*P-heterogeneity*	*0*.*78*			*0*.*90*			*1*.*00*		
**≥20 and <60 years**									
Enteric	22	11.3	1.94 (1.22–2.94)*	17	5.5	3.10 (1.81–4.97)***	4	4.9	0.82 (0.22–2.09)
Septicemic	0	0.24	0.00 (0.00–15.69)	0	0.1	0.00 (0.00–32.62)	0	0.1	0.00 (0.00–36.14)
Other†	0	0.26	0.00 (0.00–14.10)	0	0.1	0.00 (0.00–28.91)	0	0.1	0.00 (0.00–32.23)
*P-heterogeneity*	*1*.*00*			*1*.*00*			*1*.*00*		
**Age at infection**	**Obs**	**Exp**	**SIR (95% CI)**	**Obs§**	**Exp**	**SIR (95% CI)**	**Obs§**	**Exp**	**SIR (95% CI)**
20–39 years	4	1.4	2.95 (0.80–7.56)	1	0.75	1.34 (0.03–7.45)	2	0.6	3.33 (0.50–7.01)
40–59 years	18	10.5	1.64 (1.72–2.72)*	16	4.9	3.22 (1.84–5.23)***	2	4.5	0.45 (0.05–1.61)
60–69 years	10	10.0	1.00 (0.48–1.83)	9	5.31	1.70 (0.78–3.22)	1	4.1	0.25 (0.01–1.37)
≥70 years	11	11.3	0.97 (0.48–1.74)	7	6.71	1.04 (0.42–2.15)	4	4.3	0.94 (0.26–2.40)
*P-heterogeneity*	*0*.*13*			*0*.*07*			*0*.*12*		
*P-trend*	*0*.*03*			*0*.*02*			*0*.*40*		

*p-value <0.05

**p-value <0.01

***p-value <0.001.

§3 colon cancer cases were excluded from the colon subsite-specific analysis as they had cancer involving both the ascending/transverse and descending/sigmoid regions of the colon.

†*Salmonella* isolated from urinary tract or wound infections.

Risk of colon cancer as a whole and per subsite following an infection with *Salmonella* Enteritidis stratified by age at infection, type of infection, and by follow-up time for patients of all ages (≥20 years) and for those <60 years at infection. Observed (Obs) and expected (Exp) numbers of cancers, standardized incidence ratio (SIR) with 95% confidence interval (CI), test of SIR for heterogeneity.

The time to onset of colon cancer after *Salmonella* infection is unknown and we therefore repeated the analyses considering the time at risk to start at 4, 7 or 10 years post-infection. The increased SIRs of cancer in the ascending/transverse colon after *Salmonella* (Enteritidis) infection occurring between 20 and 60 years of age remained significant over the analyses (S2–S7 Tables).

### Within-cohort comparisons and analysis of pathology records

Joint Cox analysis showed that, within the cohort (i.e. among salmonellosis cases only), the risk of colon cancer did not differ significantly by sex, serovar, age at infection, type of infection or SES, and that the HRs of cancer in the ascending/transverse colon *vs*. descending/sigmoid colon within the cohort were not significantly different, possibly because of low statistical power (S8 Table).

From the National Pathology Records data base PALGA, information was obtained on IBD, mutations in the Ras/Raf/Mapk pathway, MSI, tumor stage and differentiation for 65 colon cancer patients with prior salmonellosis and this was compared with 194 age- and gender-matched colon cancer patients without a reported history of *Salmonella* infection. Colon cancer patients with reported salmonellosis more frequently had IBD (7.7%) than colon cancer controls (2.1%, RR 3.1; 95%CI 1.2–8.0) (Table 4 and S9 Table).

**Table 4:** Binomial regression analysis of tumor pathology records.

	Colon cancer (overall)			Ascending & transverse colon		
	Salm+	Sal-	RR (95% CI)§	Salm+	Salm-	RR (95% CI)§
**Inflammatory bowel disease**						
No	60	190	Reference	44	141	Reference
Yes	5	4	2.75 (1.48–5.10)	4	3	2.95 (1.47–5.92)
**Tumor stage**						
0-I	15	23	Reference	12	17	Reference
II	12	54	0.40 (0.21–0.78)**	11	43	0.43 (0.21–0.88)*
III	25	74	0.55 (0.32–0.94)*	15	51	0.45 (0.24–0.87)*
IV	11	42	0.49 (0.25–0.94)*	9	32	0.49 (0.24–1.00)*
Unknown	2	1	1.80 (0.71–4.59)	1	1	1.29 (0.28–5.90)

§ All estimates are corrected for the matching variables gender and age at cancer diagnosis. Retained in the models are only those factors that were significantly associated with the outcome (i.e. having experienced reported *Salmonella* infection) or that changed the RRs of the other covariates >10% when removed from the model. Besides inflammatory bowel disease (IBD) and tumor stage (based on TNM classification), variables tested for association in the analysis were presence/absence of microsatellite instability (MSI), genetic predisposition (mutations in the Ras/Raf/Mapk pathway) and tumor differentiation (S9 Table).

*p-value <0.05

**p-value <0.01

***p-value <0.001.

Output of the multivariable binomial regression analysis predicting reported *Salmonella* infection among patients with colon cancer as a function of genetic and tumor pathological factors of the colon cancer patients using a three times larger gender- and age-matched colon cancer control group without reported *Salmonella* infection. Colon cancer cases per colon subsite affected with (Salm+) and without (Salm-) a reported *Salmonella* infection, risk ratio (RR) and 95% confidence interval (CI).

IBD patients represented a small group (*n* = 5) of all patients with a salmonellosis history and these were diagnosed with IBD before salmonellosis (Table 5). Colon cancer patients with a history of *Salmonella* infection were also significantly more likely to present with lower stage tumors (Table 4 and S9 Table). Occurrence of genetic predisposition, MSI and undifferentiated cancer phenotypes did not differ between colon cancer patients with and without reported history of salmonellosis (S9 Table).

**Table 5:** Year of diagnosis IBD patients.

Year IBD diagnosis	Year *Salmonella* infection	Year cancer diagnosis	*Salmonella* serovar diagnosed
2000	2000	2013	Enteritidis
2000	2003	2005	Enteritidis
2005	2012	2015	Enteritidis
2001	2002	2015	Typhimurium
2000	2007	2015	Other

Year of diagnosis for inflammatory bowel disease (IBD), *Salmonella* infection, and colon cancer in patients with a history of *Salmonella* infection. The data are extracted from the pathology records (via PALGA) of the selected patient group.

## Discussion

To assess whether *Salmonella* infection constitutes a risk factor for colon cancer, we compared the incidence of colon cancer among Dutch residents with a history of (severe) *Salmonella* infection to that in the general Dutch population (i.e. the baseline reference incidence). Moreover, we examined potential effects of gender, age, latency, serovar, type of infection, SES, IBD, genetic predisposition and tumor pathological features on the association between *Salmonella* infection and colon cancer.

We observed an increased risk of cancer in the ascending and transverse parts of the colon in patients with a reported history of *Salmonella* infection, with the highest risk for those patients aged <60 years at the time of infection, as compared to the general population. This increased risk was most strongly related to infection with *S*. Enteritidis. While the terminal ileum is where *Salmonella* mainly resides [36], the ascending and transverse portions of the colon are colon subsites most exposed to *Salmonella* bacteria leaving the ileum. Various factors may then contribute to carcinogenesis in the colon, including inflammation, dysbiosis and continuous growth of epithelial cells with a risk of acquiring pretransforming mutations. Consequently, pre-malignant forms (i.e. polyps) of colon cancer are often observed, especially at higher age. If *Salmonella* infection provides one step in the multistep process resulting in cancer[13, 18], infection of premalignant colon polyps could induce full transformation.

We also assessed several potential confounders, such as SES, IBD, genetic predisposition and tumor pathological features. SES is a proxy for many factors, including diet, general health status, smoking behavior, physical activity and obesity [37], which did not contribute significantly to salmonellosis-associated colon cancer risk. Only IBD occurrence differed significantly between colon cancer patients with and without reported *Salmonella* infection, but the small number of IBD patients (*n* = 5) relative to the total number of colon cancer patients with a history of salmonellosis for which IBD status was known (*n* = 65), suggests that IBD contribution is minimal. Yet, IBD may predispose to a more intense or longer periods of *Salmonella* infection, thereby further increasing the risk of developing colon cancer.

To date, several risk factors for colon cancer have been identified. These include IBD [26, 27] and genetic mutations [28, 29]. Yet colon cancer incidence increases over the years as a function of largely unknown risk factors [30, 31]. Whether colon cancer is pure ‘bad genetic luck’ or whether external factors like a protein- and fat-rich diet and sedentary lifestyle are essential contributors, is still unclear [29]. Fruit, for example, contains anti-oxidants that may reduce radical formation and DNA mutations leading to colon cancer [38]. Dietary calcium, vitamin D and folate can also modulate colon carcinogenesis [39]. Moreover, both chronic inflammation (e.g. IBD) and diet may affect the gut microbiome, and some microorganisms may secrete mutating compounds directly [40] or indirectly by modulating the immune response in the colon [41]. While genetic predisposition (SIR 2.2–3.9) and IBD (SIR 2.6–2.8) constitute the major reported factors, alcohol abuse, obesity, and consumption of red and processed meat (SIR 1.2) contribute more marginally. Obviously, consumption of (undercooked) meat is also a risk factor for acquiring *Salmonella* infection [42], which may also contribute to this association with colon cancer. Moreover, cancer is fueled by deregulation of signaling pathways in control of cellular growth and proliferation, and these pathways are sometimes targeted by bacteria to establish infection [15]. Trivializing a major foodborne pathogen like *Salmonella* as a mere causative agent of gastroenteritis would therefore ignore its known potential to manipulate host cell signaling pathways in their infectious cycle [13, 17] in a way that would also contribute a step in the cancer formation cascade. The role of bacterial effectors in host cell cell biology is complex. For example, *Salmonella* protein AvrA is injected in host cells by the Type Three Secretion System (TTSS) to suppress apoptosis particularly in the context of enteropathogenic salmonellosis to prolong intracellular bacterial survival [43][44]. This would explain why infection invasiveness does not associate to transformation, as significantly increased SIRs for colon cancer were only observed for enteric, and not for septicemic, infections. AvrA-expressing *Salmonella* bacteria also increase the number of stem cells and proliferative cells in infected intestinal mucosa by activating the Wnt/β-catenin pathway [45], with *Salmonella* being confirmed to persist in the colon for up to 45 weeks, not only in epithelial cells on the colonic luminal surface and base of the crypts, but also in invading colorectal tumors whose incidence has been reported to be significantly increased in AvrA+ *vs*. AvrA− *Salmonella* infected mice [18]. This is not only the case in mice and AvrA is also detected in inflamed colorectal tumors and its precursor lesions in human clinical specimens [19], providing at least one plausible mechanistic explanation as to how *Salmonella* infections could contribute to colon cancer development.

We observed that the tumors of patients with a history of *Salmonella* were mainly of low grade. It is unclear why, but one option is that, by virtue of their history, these tumors are different from those developing without a contribution from *Salmonella* infection, as observed for the gallbladder carcinomas with a history of *S*. Typhi infection [13]. The tumors associated with salmonellosis may have started from already pre-transformed cells, and while transformation from a pre-malignant to a malignant (full and advanced carcinoma) state would usually take around 4 years, cell, organoid and mouse experiments suggest that *Salmonella* infection can accelerate this transformation considerably [13]. Yet, it is unknown how long this transformation would take in humans. For this reason, we repeated the analyses setting the start of the follow-up period at 1, 4, 7 and 10 years post-infection, which all yielded similar increased risks of colon cancer after *S*.Enteritidis infection. It is possible that the patients diagnosed with colon cancer within 4 years from *Salmonella* infection had already premalignant colon polyps or adenomas whose transformation to cancer was accelerated by the *Salmonella* infection itself, thereby mimicking the situation observed under laboratory conditions [13], whereas patients diagnosed with colon cancer afterwards have had a different contribution from *Salmonella* infection, possibly including induction of the pre-transformed state itself.

*S*. Enteritidis infection showed a 3-fold increased risk of cancer in the ascending/transverse colon as compared to the general population, although it was not significantly different from the effects of the other *Salmonella* serovars when considering the salmonellosis cases only (i.e. within the cohort). There are several major differences in the epidemiology and biology of *Salmonella* serovars that could potentially explain any serovar effect on colon cancer risk. For instance, *S*. Typhimurium, unlike *S*. Enteritidis, contaminates virtually all foods of animal origin (mainly meat products), vegetables and even the environment. People are thus repeatedly exposed to *S*. Typhimurium from a young age [42] and may acquire some immunity to (severe) infection with this serovar. By contrast, *S*. Enteritidis is a highly poultry-adapted serovar whose transmission in industrialized countries like the Netherlands is almost exclusively related to consumption of raw eggs and other poultry products [42, 46], affecting mainly adults. Consequently, acquired immunity against *S*. Enteritidis is less likely to occur and infection may therefore be more severe or persistent. It is also possible that one or more of the 29 effectors specific for serovar *S*. Enteritidis would support transformation more incisively [47]. The molecular mechanisms of the different *Salmonella* serovar effects on colon tumor formation are as yet unclear.

Laboratory experiments indicate that *Salmonella* (Typhi and Typhimurium) can contribute to some steps in the multistep process to oncogenic transformation [13]. A coincidental infection of a pre-transformed cell may therefore suffice in driving cancer development. Persistent or severe *Salmonella* infections would then increase the risk of developing cancer, as the chances of infecting a pre-transformed cell are higher under these circumstances. Patients diagnosed with *Salmonella* infections and reported to public health authorities in the Netherlands are typically (severely) ill for at least 1–2 weeks. This represents only a small part of all *Salmonella* infected persons. The number of symptomatic *Salmonella* infections in the Dutch population during 1999–2015 is estimated at around 994,200 [22], approximately 35 times higher than the diagnosed cases included in this study. Most infections have a mild clinical presentation, with symptoms lasting only a few days, thus prompting no medical attention, laboratory testing and reporting. Consequently, the group of colon cancer patients without a reported *Salmonella* infection may have included patients with undiagnosed, milder *Salmonella* infections, with unknown contribution to colon cancer. Of the circa 93.8 million cases of salmonellosis estimated to occur annually worldwide, a significant number is attributable to *S*. Enteritidis [21], which may thus in principle contribute to colon cancer formation in a significant number of persons. The *Salmonella* infections included in this study represent severely infected patients diagnosed after long-lasting illness, including severe symptoms like bloody diarrhea, dehydration, etc. requiring medical attention and even hospitalization. The potential contribution to colon cancer of the larger number of unreported (mild) *Salmonella* infections occurring in the population is implicitly included in the baseline colon cancer incidence used for comparison, so the increased risks identified here can be considered a conservative estimate. This is further suggested by serology studies suggesting that subjects with increased *Salmonella* FliC antibody titers may also have an increased risk of colorectal cancer [48].

This study was inspired by an experiment showing that APC+/- mice infected with *S*. Typhimurium developed colon cancer following infection [13]. This does not mean that the epidemiological results reported here can be explained by these mouse experiments. Indeed, the pathogenesis of salmonellosis in mice differs from humans. Yet, by virtue of the ability of *Salmonella* bacteria to manipulate host cell signaling pathways in a way that would promote their transformation, one of the possible outcomes of *Salmonella* infections could in fact be similar regardless of the host in question. Human are not germ-line APC heterozygous, which would decrease risk of transformation by *Salmonella*. Yet, humans have a considerably larger colon than mice, and more cells–including pretransformed cells- can be infected. It was unclear how these factors weight in the ultimate outcome of infection and transformation, but an increased risk of colon cancer is observed at least following *S*.Enteritidis infection. While the results of this epidemiological study suggest that *Salmonella* infection increases colon cancer risk and mechanistic biological explanations are available [13, 19], the exact cascade of events leading to such increased risk needs to be further disentangled.

In conclusion, to a brief list of bacterial pathogens contributing to cancer, including so far *S*. Typhi infection in association with gallbladder cancer (SIR 7–11) [10, 11, 13] and *H*. *pylori* infection with gastric cancer (SIR 5.8) [24], our data suggest that the major foodborne pathogen *Salmonella* Enteritidis in association with colon cancer may be added. While we have described a molecular mechanism for the role of *Salmonella* in gallbladder carcinoma formation in man and colon cancer formation in mice [13], these data have to be confirmed in the human colon cancer samples in patients with a history of severe *Salmonella* infection. Independent confirmation of the relationship between *S*.Enteritidis and colon cancer in independent data sets will further strengthen these points, as required to convince health authorities in allowing early participation of patients diagnosed for this pathogen into National Colon Cancer Screening Programs.

## Supporting information

S1 Table Description of all patients with a reported Salmonella infection in the Netherlands during 1999–2015 (n = 28,117). (DOCX)

S2 Table Colon cancer risk by gender and age at *Salmonella* infection, with time at risk starting 4 years after infection. (DOCX)

S3 Table Colon cancer risk by follow-up, *Salmonella* serovar and type of infection, with time at risk starting 4 years after infection. (DOCX)

S4 Table Colon cancer risk by gender and age at *Salmonella* infection with time at risk starting 7 years after infection. (DOCX)

S5 Table Colon cancer risk by follow-up, *Salmonella* serovar and type of infection, with time at risk starting 7 years after infection. (DOCX)

S6 Table Colon cancer risk by gender and age at *Salmonella* infection with time at risk starting 10 years after infection. (DOCX)

S7 Table Colon cancer risk by follow-up, *Salmonella* serovar and type of infection, with time at risk starting 10 years after infection. (DOCX)

S8 Table Joint Cox proportional hazards analysis of colon cancer within the cohort. (DOCX)

S9 Table Outputs from the univariate binomial regression analysis of pathology records. (DOCX)

S1 Fig Incidence of colon cancer according to age in the Dutch population between 1999 and 2016 (in bars) and projected standardized incidence ratio (SIR) and 95% confidence interval (CI) for colon cancer among those infected in the same decade of life with *Salmonella* (dots). (DOCX)

S2 Fig Cumulative incidence of colon cancer by attained age in patients with reported *Salmonella* infection. (DOCX)

S3 Fig Cumulative incidence of cancer in the descending and sigmoid parts of the colon. (DOCX)
