# Supplementary material for: Increased colon cancer risk after severe Salmonella infection
Source: PLoS One. 2018 Jan 17;13(1):e0189721. doi: 10.1371/journal.pone.0189721 (PMC5771566; doi:10.1371/journal.pone.0189721)
Supplement: S1 Table — (DOCX) [file pone.0189721.s001.docx]

**S1 Table:** **Description of all patients with a reported *Salmonella* infection in the Netherlands during 1999-2015 (*n*=28,117).**

|  | **Typhimurium** | **Enteritidis** | **Other** |
| --- | --- | --- | --- |
| **Gender** |  |  |  |
| Male | 4713 (34.42%) | 4945 (36.12%) | 4033 (29.46%) |
| Female | 4693 (32.53%) | 5176 (35.88%) | 4557 (31.59%) |
| **Age at infection** |  |  |  |
| ≤19 years | 5179 (43.13%) | 4418 (36.79%) | 2411 (20.08%) |
| 20-39 years | 1416 (23.10%) | 2379 (38.82%) | 2334 (38.08%) |
| 40-49 years | 442 (19.95%) | 931 (42.03%) | 842 (38.01%) |
| 50-59 years | 513 (21.15%) | 909 (37.47%) | 1004 (41.38%) |
| 60-69 years | 660 (29.93%) | 685 (31.07%) | 860 (39.00%) |
| ≥70 years | 1196 (38.16%) | 799 (25.49%) | 1139 (36.34%) |
| **Follow-up period (years at risk)** |  |  |  |
| ≤7 | 4176 (35.21%) | 3089 (26.04%) | 4596 (38.75%) |
| >7 | 5230 (32.17%) | 7032 (43.26%) | 3994 (24.57%) |
| **Socioeconomic status** |  |  |  |
| Low | 576 (32.73%) | 715 (40.63%) | 469 (26.65%) |
| Intermediate | 5458 (35.87%) | 5168 (33.96%) | 4592 (30.17%) |
| High | 2601 (29.25%) | 3451 (38.81%) | 2840 (31.94%) |
| Unknown | 771 (34.31%) | 787 (35.02%) | 689 (30.66%) |
| **Type of the infection (isolation sample)** |  |  |  |
| Enteric (feces) | 8667 (33.95%) | 9463 (37.07%) | 7397 (28.98%) |
| Septicemic (blood, liquor) | 289 (24.27%) | 319 (26.78%) | 583 (48.95%) |
| Other (urine, wound) | 450 (32.17%) | 339 (24.23%) | 610 (43.60%) |
