# Supplementary material for: Increased colon cancer risk after severe Salmonella infection
Source: PLoS One. 2018 Jan 17;13(1):e0189721. doi: 10.1371/journal.pone.0189721 (PMC5771566; doi:10.1371/journal.pone.0189721)
Supplement: S2 Table — (DOCX) [file pone.0189721.s002.docx]

**S2 Table: Colon cancer risk by gender and age at *Salmonella* infection, with time at risk starting 4 years after infection.**

Risk of colon cancer as a whole and per subsite by gender and age at *Salmonella* infection for patients of all ages (≥20 years) and for those <60 years at infection, with time at risk starting 4 years after infection. Observed (Obs) and expected (Exp) numbers of cancers, standardized incidence ratio (SIR) with 95% confidence interval (CI), test of SIR for heterogeneity and trend.

| **Gender** | **Colon cancer (overall)** | | | | | **Ascending & transverse colon** | | | | | | **Descending & sigmoid colon** | | | | | |  |
| --- | --- | --- | --- | --- | --- | --- | --- | --- | --- | --- | --- | --- | --- | --- | --- | --- | --- | --- |
| **All ages ≥20 years** | **Obs** | **Exp** | | **SIR (95% CI)** | | **Obs§** | | **Exp** | | **SIR (95% CI)** | | **Obs§** | | **Exp** | | **SIR (95% CI)** | |  |
| Overall | 63 | 53.9 | | 1.17 (0.90-1.50) | | 45 | | 28.9 | | 1.56 (1.14-2.08)** | | 16 | | 21.4 | | 0.75 (0.43-1.22) | |  |
| Male | 34 | 24.2 | | 1.41 (0.97-1.96) | | 23 | | 11.9 | | 1.92 (1.22-2.88)** | | 9 | | 9.6 | | 0.94 (0.43-1.78) | |  |
| Female | 29 | 29.7 | | 0.98 (0.65-1.40) | | 22 | | 16.9 | | 1.32 (0.82-1.97) | | 7 | | 11.8 | | 0.60 (0.24-1.23) | |  |
| *P-heterogeneity* | *0.15* | | |  | | *0.19* | | | |  | | *0.37* | | | |  | |  |
| **≥20 and <60 years** |  | | | | |  | | | | | |  | | | | | |  |
| Overall | 29 | 20.6 | | 1.41 (0.94-2.03) | | 20 | | 9.9 | | 2.02 (1.23-3.12)** | | 8 | | 8.7 | | 0.92 (0.40-1.81) | |  |
| Male | 17 | 10.5 | | 1.62 (0.94-2.59) | | 11 | | 4.7 | | 2.33 (1.16-4.17)* | | 5 | | 4.4 | | 1.13 (0.37-2.63) | |  |
| Female | 12 | 10.1 | | 1.19 (0.62-2.09) | | 9 | | 5.2 | | 1.74 (0.79-3.29) | | 3 | | 4.3 | | 0.70 (0.14-2.05) | |  |
| *P-heterogeneity* | *0.42* | | |  | | *0.51* | | | |  | | *0.52* | | | |  | |  |
| **Age at infection** | **Obs** | | **Exp** | | **SIR (95% CI)** | | **Obs§** | | **Exp** | | **SIR (95% CI)** | | **Obs§** | | **Exp** | | **SIR (95% CI)** | |
| 20-39 years | 4 | | 2.1 | | 1.92 (0.52-4.91)* | | 3 | | 1.0 | | 2.95 (0.61-8.61) | | 1 | | 1.0 | | 1.00 (0.03-5.57) | |
| 40-49 years | 8 | | 5.2 | | 1.54 (0.66-3.04) | | 5 | | 2.5 | | 2.04 (0.66-4.75) | | 2 | | 2.4 | | 0.83 (0.10-3.01) | |
| 50-59 years | 17 | | 13.0 | | 1.30 (0.76-2.09) | | 12 | | 6.3 | | 1.95 (1.01-3.41)* | | 5 | | 5.2 | | 0.95 (0.31-2.23) | |
| 60-69 years | 22 | | 16.8 | | 1.31 (0.82-1.98) | | 17 | | 9.0 | | 1.89 (1.10-3.02)* | | 4 | | 6.6 | | 0.61 (0.17-1.56) | |
| ≥70 years | 12 | | 16.5 | | 0.73 (0.38-1.27) | | 8 | | 10.0 | | 0.80 (0.35-1.58) | | 4 | | 6.1 | | 0.66 (0.18-1.68) | |
| *P-heterogeneity* | *0.43* | | | |  | | *0.28* | | | |  | | *0.97* | | | |  | |
| *P-trend* | *0.07* | | | |  | | *0.06* | | | |  | | *0.58* | | | |  | |

*p-value <0.05; **p-value <0.01; ***p-value <0.001. §2 colon cancer cases were excluded from the colon subsite-specific analysis as they had cancer involving both the ascending/transverse and descending/sigmoid regions of the colon.
