# Supplementary material for: Increased colon cancer risk after severe Salmonella infection
Source: PLoS One. 2018 Jan 17;13(1):e0189721. doi: 10.1371/journal.pone.0189721 (PMC5771566; doi:10.1371/journal.pone.0189721)
Supplement: S3 Table — (DOCX) [file pone.0189721.s003.docx]

**S3 Table: Colon cancer risk by follow-up, *Salmonella* serovar and type of infection, with time at risk starting 4 years after infection.**

Risk of colon cancer as a whole and per subsite by follow-up time, infecting *Salmonella* serovar and type of infection for patients of all ages (≥20 years) and for those <60 years at infection, with time at risk starting 4 years after infection. Observed (Obs) and expected (Exp) numbers of cancers, standardized incidence ratio (SIR) with 95% confidence interval (CI), test of SIR for heterogeneity.

| **Follow-up time**  **(years at risk)** | **Colon cancer (overall)** | | | | **Ascending & transverse colon** | | | | **Descending & sigmoid colon** | | | |
| --- | --- | --- | --- | --- | --- | --- | --- | --- | --- | --- | --- | --- |
| **All ages ≥20 years** | **Obs** | | **Exp** | **SIR (95% CI)** | **Obs§** | | **Exp** | **SIR (95% CI)** | **Obs§** | | **Exp** | **SIR (95% CI)** |
| 4-7 years | 22 | | 20.3 | 1.08 (0.68-1.64) | 17 | | 11.0 | 1.42 (0.90-2.48) | 4 | | 8.3 | 0.48 (0.13-1.24) |
| >7 years | 41 | | 33.6 | 1.22 (0.88-1.66) | 28 | | 17.9 | 1.56 (1.04-2.26)* | 12 | | 13.1 | 0.92 (0.47-1.60) |
| *P-heterogeneity* | *0.87* | | |  | *0.48* | | |  | *0.32* | | |  |
| **≥20 and <60 years** |  | |  |  |  | |  |  |  | |  |  |
| 4-7 years | 5 | | 5.2 | 0.96 (0.31-2.23) | 5 | | 2.6 | 1.94 (0.63-4.52) | 0 | | 2.4 | 0.00 (0.00-1.54) |
| >7 years | 24 | | 15.3 | 1.57 (1.00-2.33) | 15 | | 7.3 | 2.05 (1.15-3.38)* | 8 | | 6.3 | 1.27 (0.55-2.49) |
| *P-heterogeneity* | *0.31* | | |  | *0.92* | | |  | *0.97* | | |  |
| ***Salmonella* serovar** | **Colon cancer (overall)** | | | | **Ascending & transverse colon** | | | | **Descending & sigmoid colon** | | | |
| **All ages ≥20 years** | **Obs** | | **Exp** | **SIR (95% CI)** | **Obs§** | | **Exp** | **SIR (95% CI)** | **Obs§** | | **Exp** | **SIR (95% CI)** |
| Typhimurium | 11 | | 12.3 | 0.87 (0.43-1.55) | 5 | | 6.9 | 0.72 (0.24-1.69) | 5 | | 4.9 | 1.01 (0.33-2.36) |
| Enteritidis | 31 | | 24.2 | 1.28 (0.87-1.82) | 26 | | 12.9 | 2.02 (1.32-2.96)** | 5 | | 9.6 | 0.52 (0.17-1.21) |
| Other | 21 | | 17.1 | 1.23 (0.76-1.88) | 14 | | 9.1 | 1.54 (0.84-2.58) | 6 | | 6.8 | 0.89 (0.33-1.93) |
| *P-heterogeneity* | *0.52* | | |  | *0.10* | | |  | *0.53* | | |  |
| **≥20 and <60 years** |  | | | |  | | | |  | | | |
| Typhimurium | 2 | | 3.7 | 0.53 (0.07-1.93) | 1 | | 1.8 | 0.55 (0.02-3.06) | 1 | | 1.6 | 0.63 (0.02-3.53) |
| Enteritidis | 16 | | 9.9 | 1.62 (0.93-2.63) | 13 | | 4.8 | 2.73 (1.46-4.67)** | 3 | | 4.2 | 0.71 (0.15-2.09) |
| Other | 11 | | 6.9 | 1.59 (0.79-2.84) | 6 | | 3.3 | 1.80 (0.66-3.93) | 4 | | 2.9 | 1.36 (0.37-3.48) |
| *P-heterogeneity* | *0.32* | | |  | *0.25* | | |  | *0.63* | | |  |
| **Type of infection** | **Colon cancer (overall)** | | | | **Ascending & transverse colon** | | | | **Descending & sigmoid colon** | | | |
| **All ages ≥20 years** | **Obs** | **Exp** | | **SIR (95% CI)** | **Obs§** | **Exp** | | **SIR (95% CI)** | **Obs§** | **Exp** | | **SIR (95% CI)** |
| Enteric | 56 | 48.0 | | 1.16 (0.88-1.51) | 41 | 25.7 | | 1.60 (1.15-2.17)** | 13 | 19.1 | | 0.68 (0.36-1.17) |
| Septicemic | 5 | 2.3 | | 2.20 (0.71-5.13) | 2 | 1.2 | | 1.62 (0.20-5.84) | 3 | 0.9 | | 3.36 (0.69-9.81) |
| Other† | 2 | 3.6 | | 0.56 (0.07-2.01) | 2 | 2.0 | | 1.00 (0.12-3.62) | 0 | 1.4 | | 0.00 (0.00-2.66) |
| *P-heterogeneity* | *0.22* | | |  | *0.81* | | |  | *0.05* | | |  |
| **≥20 and <60 years** |  | | |  |  | | |  |  | | |  |
| Enteric | 28 | 19.1 | | 1.47 (0.97-2.19) | 20 | 9.2 | | 2.17 (1.33-3.36)** | 7 | 8.1 | | 0.86 (0.35-1.78) |
| Septicemic | 1 | 0.6 | | 1.69 (0.04-9.43) | 0 | 0.3 | | 0.00 (0.00-13.13) | 1 | 0.3 | | 3.92 (0.10-21.82) |
| Other† | 0 | 0.9 | | 0.00 (0.00-4.22) | 0 | 0.4 | | 0.00 (0.00-8.83) | 0 | 0.4 | | 0.00 (0.00-10.10) |
| *P-heterogeneity* | *0.99* | | |  | *1.00* | | |  | *0.37* | | |  |

*p-value <0.05; **p-value <0.01; ***p-value <0.001. §2 colon cancer cases were excluded from the colon subsite-specific analysis as they had cancer involving both the ascending/transverse and descending/sigmoid regions of the colon. †*Salmonella* isolated from urinary tract or wound infections.
