# Supplementary material for: Increased colon cancer risk after severe Salmonella infection
Source: PLoS One. 2018 Jan 17;13(1):e0189721. doi: 10.1371/journal.pone.0189721 (PMC5771566; doi:10.1371/journal.pone.0189721)
Supplement: S4 Table — (DOCX) [file pone.0189721.s004.docx]

**S4 Table: Colon cancer risk by gender and age at *Salmonella* infection with time at risk starting 7 years after infection.**

Risk of colon cancer as a whole and per subsite by gender and age at *Salmonella* infection for patients of all ages (≥20 years) and for those <60 years at infection, with time at risk starting 7 years after infection. Observed (Obs) and expected (Exp) numbers of cancers, standardized incidence ratio (SIR) with 95% confidence interval (CI), test of SIR for heterogeneity and trend.

| **Gender** | **Colon cancer (overall)** | | | | | **Ascending & transverse colon** | | | | | | **Descending & sigmoid colon** | | | | | |  |
| --- | --- | --- | --- | --- | --- | --- | --- | --- | --- | --- | --- | --- | --- | --- | --- | --- | --- | --- |
| **All ages ≥20 years** | **Obs** | **Exp** | | **SIR (95% CI)** | | **Obs§** | | **Exp** | | **SIR (95% CI)** | | **Obs§** | | **Exp** | | **SIR (95% CI)** | |  |
| Overall | 41 | 33.6 | | 1.22 (0.88-1.66) | | 28 | | 17.9 | | 1.56 (1.04-2.26)* | | 12 | | 13.1 | | 0.92 (0.47-1.60) | |  |
| Male | 24 | 15.6 | | 1.54 (0.99-2.29) | | 16 | | 7.5 | | 2.14 (1.22-3.47)** | | 7 | | 6.2 | | 1.13 (0.45-2.32) | |  |
| Female | 17 | 18.0 | | 0.94 (0.55-1.51) | | 12 | | 10.5 | | 1.15 (0.59-2.01) | | 5 | | 6.9 | | 0.73 (0.24-1.69) | |  |
| *P-heterogeneity* | *0.12* | | |  | | *0.10* | | | |  | | *0.45* | | | |  | |  |
| **≥20 and <60 years** |  | | | | |  | | | | | |  | | | | | |  |
| Overall | 24 | 15.3 | | 1.56 (1.01-2.33)* | | 15 | | 7.3 | | 2.05 (1.15-3.38)* | | 8 | | 6.3 | | 1.27 (0.55-2.49) | |  |
| Male | 15 | 7.9 | | 1.89 (1.06-3.11)* | | 9 | | 3.5 | | 2.59 (1.19-4.92)* | | 5 | | 3.3 | | 1.52 (0.49-3.55) | |  |
| Female | 9 | 7.4 | | 1.22 (0.57-2.31) | | 6 | | 3.9 | | 1.56 (0.56-3.39) | | 3 | | 3.0 | | 0.99 (0.20-2.89) | |  |
| *P-heterogeneity* | *0.30* | | |  | | *0.36* | | | |  | | *0.56* | | | |  | |  |
| **Age at infection** | **Obs** | | **Exp** | | **SIR (95% CI)** | | **Obs**§ | | **Exp** | | **SIR (95% CI)** | | **Obs**§ | | **Exp** | | **SIR (95% CI)** | |
| 20-39 years | 4 | | 1.7 | | 2.42 (0.66-6.19) | | 3 | | 0.8 | | 3.77 (0.78-11.01) | | 1 | | 0.8 | | 1.26 (0.03-7.01) | |
| 40-49 years | 7 | | 4.0 | | 1.74 (0.70-3.58) | | 4 | | 2.0 | | 2.11 (0.57-5.39) | | 2 | | 1.8 | | 1.09 (0.13-3.92) | |
| 50-59 years | 13 | | 9.4 | | 1.38 (0.73-2.36) | | 8 | | 4.4 | | 1.81 (0.78-3.57) | | 5 | | 3.6 | | 1.38 (0.45-3.22) | |
| 60-69 years | 14 | | 10.7 | | 1.31 (0.71-2.19) | | 12 | | 5.9 | | 2.04 (1.06-3.57)* | | 2 | | 4.1 | | 0.49 (0.06-1.78) | |
| ≥70 years | 3 | | 7.6 | | 0.40 (0.08-1.16) | | 1 | | 4.7 | | 0.21 (0.01-1.18) | | 2 | | 2.7 | | 0.74 (0.10-2.67) | |
| *P-heterogeneity* | *0.31* | | | |  | | *0.30* | | | |  | | *0.79* | | | |  | |
| *P-trend* | *0.02* | | | |  | | *0.03* | | | |  | | *0.41* | | | |  | |

*p-value <0.05; **p-value <0.01; ***p-value <0.001. §1 colon cancer case was excluded from the colon subsite-specific analysis as it had cancer involving both the ascending/transverse and descending/sigmoid regions of the colon.
