# Supplementary material for: Increased colon cancer risk after severe Salmonella infection
Source: PLoS One. 2018 Jan 17;13(1):e0189721. doi: 10.1371/journal.pone.0189721 (PMC5771566; doi:10.1371/journal.pone.0189721)
Supplement: S5 Table — (DOCX) [file pone.0189721.s005.docx]

**S5 Table: Colon cancer risk by follow-up, *Salmonella* serovar and type of infection, with time at risk starting 7 years after infection.**

Risk of colon cancer as a whole and per subsite by follow-up time, infecting *Salmonella* serovar and type of infection for patients of all ages (≥20 years) and for those <60 years at infection, with time at risk starting 7 years after infection. Observed (Obs) and expected (Exp) numbers of cancers, standardized incidence ratio (SIR) with 95% confidence interval (CI), test of SIR for heterogeneity.

| **Follow-up time**  **(years at risk)** | **Colon cancer (overall)** | | | | **Ascending & transverse colon** | | | | **Descending & sigmoid colon** | | | |
| --- | --- | --- | --- | --- | --- | --- | --- | --- | --- | --- | --- | --- |
| **All ages ≥20 years** | **Obs** | | **Exp** | **SIR (95% CI)** | **Obs§** | | **Exp** | **SIR (95% CI)** | **Obs§** | | **Exp** | **SIR (95% CI)** |
| 7-10 years | 18 | | 15.5 | 1.16 (0.69-1.84) | 12 | | 8.3 | 1.44 (0.74-2.51) | 6 | | 6.2 | 0.96 (0.35-2.10) |
| >10 years | 23 | | 18.1 | 1.27 (0.81-1.91) | 16 | | 9.6 | 1.68 (0.95-2.71) | 6 | | 6.9 | 0.87 (0.32-1.90) |
| *P-heterogeneity* | *0.77* | | |  | *0.69* | | |  | *0.86* | | |  |
| **≥20 and <60 years** |  | |  |  |  | |  |  |  | |  |  |
| 7-10 years | 7 | | 5.8 | 1.21 (0.49-2.50) | 4 | | 2.8 | 1.43 (0.39-3.67) | 3 | | 2.5 | 1.20 (0.25-3.50) |
| >10 years | 17 | | 9.6 | 1.78 (1.04-2.85) | 11 | | 4.5 | 2.43 (1.21-4.34)* | 5 | | 3.8 | 1.31 (0.43-3.06) |
| *P-heterogeneity* | *0.39* | | |  | *0.37* | | |  | *0.90* | | |  |
| ***Salmonella* serovar** | **Colon cancer (overall)** | | | | **Ascending & transverse colon** | | | | **Descending & sigmoid colon** | | | |
| **All ages ≥20 years** | **Obs** | | **Exp** | **SIR (95% CI)** | **Obs§** | | **Exp** | **SIR (95% CI)** | **Obs§** | | **Exp** | **SIR (95% CI)** |
| Typhimurium | 4 | | 6.9 | 0.58 (0.16-1.48) | 1 | | 3.8 | 0.27 (0.01-1.48) | 3 | | 2.7 | 1.12 (0.23-3.28) |
| Enteritidis | 22 | | 15.9 | 1.38 (0.87-2.09) | 18 | | 2.1 | 2.12 (1.26-3.36)** | 4 | | 6.2 | 0.64 (0.18-1.64) |
| Other | 15 | | 10.7 | 1.40 (0.78-2.30) | 9 | | 1.6 | 1.58 (0.72-3.00) | 5 | | 4.2 | 1.19 (0.38-2.78) |
| *P-heterogeneity* | *0.25* | | |  | *0.12* | | |  | *0.62* | | |  |
| **≥20 and <60 years** |  | | | |  | | | |  | | | |
| Typhimurium | 2 | | 2.7 | 0.75 (0.09-2.70) | 1 | | 1.3 | 0.78 (0.02-4.32) | 1 | | 1.1 | 0.91 (0.02-5.08) |
| Enteritidis | 13 | | 7.5 | 1.73 (0.92-2.96) | 10 | | 3.6 | 2.79 (1.34-5.13)*** | 3 | | 3.1 | 0.97 (0.20-2.82) |
| Other | 9 | | 5.1 | 1.75 (0.80-3.32) | 4 | | 2.4 | 1.63 (0.45-4.18) | 4 | | 2.1 | 1.89 (0.51-4.83) |
| *P-heterogeneity* | *0.52* | | |  | *0.37* | | |  | *0.63* | | |  |
| **Type of infection** | **Colon cancer (overall)** | | | | **Ascending & transverse colon** | | | | **Descending & sigmoid colon** | | | |
| **All ages ≥20 years** | **Obs** | **Exp** | | **SIR (95% CI)** | **Obs§** | **Exp** | | **SIR (95% CI)** | **Obs§** | **Exp** | | **SIR (95% CI)** |
| Enteric | 38 | 30.4 | | 1.25 (0.88-1.72) | 27 | 16.2 | | 1.67 (1.10-2.43)* | 10 | 11.9 | | 0.84 (0.40-1.55) |
| Septicemic | 2 | 1.2 | | 1.64 (0.20-5.91) | 0 | 0.7 | | 0.00 (0.00-5.58) | 2 | 0.5 | | 4.22 (0.51-15.23) |
| Other† | 1 | 2.0 | | 0.51 (0.01-2.83) | 1 | 1.1 | | 0.90 (0.02-5.03) | 0 | 0.7 | | 0.00 (0.00-5.01) |
| *P-heterogeneity* | *0.62* | | |  | *0.83* | | |  | *0.12* | | |  |
| **≥20 and <60 years** |  | | |  |  | | |  |  | | |  |
| Enteric | 23 | 14.3 | | 1.61 (1.02-2.41)* | 15 | 6.8 | | 2.19 (1.23-3.62)** | 7 | 5.9 | | 1.19 (0.48-2.45) |
| Septicemic | 1 | 0.4 | | 2.32 (0.06-12.95) | 0 | 0.2 | | 0.00 (0.00-18.42) | 1 | 0.2 | | 5.52 (0.14-30.77) |
| Other† | 0 | 0.6 | | 0.00 (0.00-6.13) | 0 | 0.3 | | 0.00 (0.00-12.96) | 0 | 0.2 | | 0.00 (0.00-15.10) |
| *P-heterogeneity* | *0.94* | | |  | *1.00* | | |  | *0.36* | | |  |

*p-value <0.05; **p-value <0.01; ***p-value <0.001. §1 colon cancer case was excluded from the colon subsite-specific analysis as it had cancer involving both the ascending/transverse and descending/sigmoid regions of the colon. †*Salmonella* isolated from urinary tract or wound infections.
