# Supplementary material for: Increased colon cancer risk after severe Salmonella infection
Source: PLoS One. 2018 Jan 17;13(1):e0189721. doi: 10.1371/journal.pone.0189721 (PMC5771566; doi:10.1371/journal.pone.0189721)
Supplement: S6 Table — (DOCX) [file pone.0189721.s006.docx]

**S6 Table: Colon cancer risk by gender and age at *Salmonella* infection with time at risk starting 10 years after infection.**

Risk of colon cancer as a whole and per subsite by gender and age at *Salmonella* infection for patients of all ages (≥20 years) and for those <60 years at infection, with time at risk starting 10 years after infection. Observed (Obs) and expected (Exp) numbers of cancers, standardized incidence ratio (SIR) with 95% confidence interval (CI), test of SIR for heterogeneity and trend.

| **Gender** | **Colon cancer (overall)** | | | | | **Ascending & transverse colon** | | | | | | **Descending & sigmoid colon** | | | | | |
| --- | --- | --- | --- | --- | --- | --- | --- | --- | --- | --- | --- | --- | --- | --- | --- | --- | --- |
| **All ages ≥20 years** | **Obs** | **Exp** | | **SIR (95% CI)** | | **Obs§** | | **Exp** | | **SIR (95% CI)** | | **Obs§** | | **Exp** | | **SIR (95% CI)** | |
| Overall | 23 | 18.1 | | 1.27 (0.81-1.91) | | 16 | | 9.6 | | 1.67 (0.95-2.71) | | 6 | | 6.9 | | 0.87 (0.32-1.90) | |
| Male | 12 | 8.8 | | 1.37 (0.71-2.39) | | 8 | | 4.0 | | 1.98 (0.86-3.90) | | 3 | | 3.5 | | 0.86 (0.18-2.52) | |
| Female | 11 | 9.3 | | 1.18 (0.59-2.11) | | 8 | | 5.5 | | 1.44 (0.62-2.84) | | 3 | | 3.4 | | 0.89 (0.18-2.59) | |
| *P-heterogeneity* | *0.72* | | |  | | *0.53* | | | |  | | *0.97* | | | |  | |
| **≥20 and <60 years** |  | | | | |  | | | | | |  | | | | | |
| Overall | 17 | 9.7 | | 1.78 (1.04-2.85) | | 11 | | 4.5 | | 2.43 (1.21-4.34)* | | 5 | | 3.8 | | 1.31 (0.43-3.06) | |
| Male | 10 | 5.0 | | 1.99 (0.96-3.66) | | 6 | | 2.1 | | 2.82 (1.31-4.00)* | | 3 | | 2.0 | | 1.47 (0.30-4.30) | |
| Female | 7 | 4.5 | | 1.54 (0.62-3.18) | | 5 | | 2.4 | | 2.08 (0.68-4.85) | | 2 | | 1.8 | | 1.13 (0.14-4.06) | |
| *P-heterogeneity* | *0.61* | | |  | | *0.61* | | | |  | | *0.77* | | | |  | |
| **Age at infection** | **Obs** | | **Exp** | | **SIR (95% CI)** | | **Obs**§ | | **Exp** | | **SIR (95% CI)** | | **Obs**§ | | **Exp** | | **SIR (95% CI)** |
| 20-39 years | 4 | | 1.1 | | 3.52 (0.96-9.01) | | 3 | | 0.5 | | 5.60 (1.15-16.36)* | | 1 | | 0.6 | | 1.82 (0.05-10.16) |
| 40-49 years | 4 | | 2.7 | | 1.47 (0.40-3.77) | | 2 | | 1.3 | | 1.58 (0.19-5.72) | | 1 | | 1.2 | | 0.84 (0.02-4.67) |
| 50-59 years | 9 | | 5.6 | | 1.62 (0.74-3.07) | | 6 | | 2.6 | | 2.32 (0.85-5.04) | | 3 | | 2.0 | | 1.48 (0.31-4.32) |
| 60-69 years | 6 | | 5.8 | | 1.04 (0.38-2.26) | | 5 | | 3.2 | | 1.55 (0.50-3.62) | | 1 | | 2.1 | | 0.48 (0.01-2.66) |
| ≥70 years | 0 | | 2.8 | | 0.00 (0.00-1.34) | | 0 | | 1.8 | | 0.00 (0.00-2.02) | | 0 | | 1.0 | | 0.00 (0.00-3.85) |
| *P-heterogeneity* | *0.94* | | | |  | | *0.97* | | | |  | | *0.91* | | | |  |
| *P-trend* | *0.01* | | | |  | | *0.04* | | | |  | | *0.24* | | | |  |

*p-value <0.05; **p-value <0.01; ***p-value <0.001. §1 colon cancer case was excluded from the colon subsite-specific analysis as it had cancer involving both the ascending/transverse and descending/sigmoid regions of the colon.
