# Supplementary material for: Increased colon cancer risk after severe Salmonella infection
Source: PLoS One. 2018 Jan 17;13(1):e0189721. doi: 10.1371/journal.pone.0189721 (PMC5771566; doi:10.1371/journal.pone.0189721)
Supplement: S7 Table — (DOCX) [file pone.0189721.s007.docx]

**S7 Table: Colon cancer risk by follow-up, *Salmonella* serovar and type of infection, with time at risk starting 10 years after infection.**

Risk of colon cancer as a whole and per subsite by follow-up time, infecting *Salmonella* serovar and type of infection for patients of all ages (≥20 years) and for those <60 years at infection, with time at risk starting 10 years after infection. Observed (Obs) and expected (Exp) numbers of cancers, standardized incidence ratio (SIR) with 95% confidence interval (CI), test of SIR for heterogeneity.

| **Follow-up time**  **(years at risk)** | **Colon cancer (overall)** | | | | **Ascending & transverse colon** | | | | **Descending & sigmoid colon** | | | |
| --- | --- | --- | --- | --- | --- | --- | --- | --- | --- | --- | --- | --- |
| **All ages ≥20 years** | **Obs** | | **Exp** | **SIR (95% CI)** | **Obs**§ | | **Exp** | **SIR (95% CI)** | **Obs**§ | | **Exp** | **SIR (95% CI)** |
| 10-13 years | 13 | | 10.1 | 1.18 (0.63-2.02) | 9 | | 5.9 | 1.53 (0.70-2.90) | 3 | | 4.2 | 0.71 (0.15-2.07) |
| >13 years | 10 | | 7.1 | 1.41 (0.67-2.59) | 7 | | 3.7 | 1.89 (0.76-3.90) | 3 | | 2.6 | 1.14 (0.24-3.33) |
| *P-heterogeneity* | *0.68* | | |  | *0.67* | | |  | *0.56* | | |  |
| **≥20 and <60 years** |  | |  |  |  | |  |  |  | |  |  |
| 10-13 years | 10 | | 5.3 | 1.88 (0.90-3.46) | 7 | | 2.5 | 2.76 (1.11-5.68)* | 2 | | 2.2 | 0.92 (0.11-3.31) |
| >13 years | 7 | | 4.2 | 1.65 (0.66-3.39) | 4 | | 2.0 | 2.01 (0.55-5.14) | 3 | | 1.6 | 1.83 (0.38-5.35) |
| *P-heterogeneity* | *0.79* | | |  | *0.61* | | |  | *0.45* | | |  |
| ***Salmonella* serovar** | **Colon cancer (overall)** | | | | **Ascending & transverse colon** | | | | **Descending & sigmoid colon** | | | |
| **All ages ≥20 years** | **Obs** | | **Exp** | **SIR (95% CI)** | **Obs**§ | | **Exp** | **SIR (95% CI)** | **Obs**§ | | **Exp** | **SIR (95% CI)** |
| Typhimurium | 1 | | 3.4 | 0.29 (0.01-1.64) | 1 | | 1.8 | 0.55 (0.01-3.04) | 0 | | 1.3 | 0.00 (0.00-2.87) |
| Enteritidis | 13 | | 8.9 | 1.46 (0.78-2.49) | 11 | | 4.7 | 2.34 (1.17-4.18)* | 2 | | 3.4 | 0.59 (0.07-2.13) |
| Other | 9 | | 5.8 | 1.56 (0.71-2.96) | 4 | | 3.0 | 1.32 (0.36-3.37) | 4 | | 2.2 | 1.82 (0.50-4.66) |
| *P-heterogeneity* | *0.28* | | |  | *0.28* | | |  | *0.43* | | |  |
| **≥20 and <60 years** |  | | | |  | | | |  | | | |
| Typhimurium | 1 | | 1.6 | 0.62 (0.02-3.46) | 1 | | 0.8 | 1.29 (0.03-7.16) | 0 | | 0.6 | 0.00 (0.00-5.70) |
| Enteritidis | 10 | | 4.8 | 2.09 (1.01-3.85)* | 8 | | 2.3 | 3.55 (1.53-6.99)** | 2 | | 1.9 | 1.05 (0.13-3.78) |
| Other | 6 | | 3.2 | 1.89 (0.69-4.12) | 2 | | 1.5 | 1.33 (0.16-4.82) | 3 | | 1.3 | 2.38 (0.49-6.95) |
| *P-heterogeneity* | *0.51* | | |  | *0.34* | | |  | *0.67* | | |  |
| **Type of infection** | **Colon cancer (overall)** | | | | **Ascending & transverse colon** | | | | **Descending & sigmoid colon** | | | |
| **All ages ≥20 years** | **Obs** | **Exp** | | **SIR (95% CI)** | **Obs** | **Exp** | | **SIR (95% CI)** | **Obs** | **Exp** | | **SIR (95% CI)** |
| Enteric | 22 | 16.6 | | 1.32 (0.83-2.00) | 16 | 8.8 | | 1.82 (1.04-2.96)* | 5 | 6.3 | | 0.79 (0.26-1.84) |
| Septicemic | 1 | 0.6 | | 1.77 (0.05-9.85) | 0 | 0.3 | | 0.00 (0.00-12.26) | 1 | 0.2 | | 4.67 (0.12-26.00) |
| Other† | 0 | 0.9 | | 0.00 (0.00-4.13) | 0 | 0.5 | | 0.00 (0.00-7.43) | 0 | 0.3 | | 0.00 (0.00-11.21) |
| *P-heterogeneity* | *0.96* | | |  | *1.00* | | |  | *0.27* | | |  |
| **≥20 and <60 years** |  | | | | | | | | | | | |
| Enteric | 16 | 8.9 | | 1.78 (1.02-2.90) | 11 | 4.3 | | 2.58 (1.29-4.62)** | 4 | 3.6 | | 1.12 (0.30-2.86) |
| Septicemic | 1 | 0.2 | | 4.06 (0.10-22.60) | 0 | 0.1 | | 0.00 (0.00-32.77) | 1 | 0.1 | | 9.90 (0.25-55.17) |
| Other† | 0 | 0.3 | | 0.00 (0.00-10.73) | 0 | 0.2 | | 0.00 (0.00-22.89) | 0 | 0.1 | | 0.00 (0.00-27.26) |
| *P-heterogeneity* | *0.73* | | |  | *1.00* | | |  | *0.15* | | |  |

*p-value <0.05; **p-value <0.01; ***p-value <0.001. §1 colon cancer case was excluded from the colon subsite-specific analysis as it had cancer involving both the ascending/transverse and descending/sigmoid regions of the colon. †*Salmonella* isolated from urinary tract or wound infections.
