# Supplementary material for: Increased colon cancer risk after severe Salmonella infection
Source: PLoS One. 2018 Jan 17;13(1):e0189721. doi: 10.1371/journal.pone.0189721 (PMC5771566; doi:10.1371/journal.pone.0189721)
Supplement: S8 Table — (DOCX) [file pone.0189721.s008.docx]

**S8 Table: Joint Cox proportional hazards analysis of colon cancer within the cohort.**

Output of the multivariable joint Cox proportional hazards regression analysis for colon cancer, accounting for death as competing risk, among patients with a reported history of *Salmonella* infection.

|  | **Colon cancer (overall)** | **Ascending & transverse colon** | **Descending & sigmoid colon** | **Ascending/transverse *vs*. descending/sigmoid colon** |
| --- | --- | --- | --- | --- |
|  | **HR (95% CI)§** | **HR (95% CI)§** | **HR (95% CI)§** |  |
| **Gender** |  |  |  |  |
| Male | Reference | Reference | Reference |  |
| Female | 0.90 (0.60-1.35) | 0.96 (0.59-1.57) | 0.84 (0.40-1.78) |  |
| *P-heterogeneity* | *0.617* | *0.880* | *0.647* | *0.764* |
| ***Salmonella* serovar** |  |  |  |  |
| Typhimurium | Reference | Reference | Reference |  |
| Enteritidis | 1.07 (0.65-1.78) | 1.54 (0.80-2.95) | 0.54 (0.22-1.36) |  |
| Other | 0.92 (0.53-1.59) | 1.12 (0.55-2.28) | 0.70 (0.28-1.73) |  |
| *P-heterogeneity* | *0.817* | *0.333* | *0.419* | *0.187* |
| **Age at *Salmonella* infection** |  |  |  |  |
| 20-39 years | Reference | Reference | Reference |  |
| 40-59 years | 1.00 (0.23-4.36) | 1.40 (0.25-7.78) | 0.19 (0.01-4.88) |  |
| ≥60 years | 0.78 (0.15-4.24) | 1.37 (0.19-10.2) | 0.10 (0.00-3.35) |  |
| *P-heterogeneity* | *0.847* | *0.927* | *0.395* | *0.439* |
| **SES** |  |  |  |  |
| Low | Reference | Reference | Reference |  |
| High | 1.19 (0.74-1.91) | 1.11 (0.62-1.99) | 1.27 (0.53-3.03) |  |
| *P-heterogeneity* | *0.464* | *0.717* | *0.590* | *0.805* |
| **Type of infection** |  |  |  |  |
| Enteric | Reference | Reference | Reference |  |
| Septicemic | 0.74 (0.27-2.03) | 0.55 (0.13-2.26) | 1.28 (0.30-5.50) |  |
| Other^†^ | 0.74 (0.29-1.83) | 0.90 (0.32-2.50) | 0.47 (0.06-3.48) |  |
| *P-heterogeneity* | *0.693* | *0.697* | *0.705* | *0.770* |

§ With attained age as time-scale and death as competing risk, accounting for left truncation by setting entry into the at-risk period one year after the age at *Salmonella* infection. *p-value <0.05; **p-value <0.01; ***p-value <0.001. SES = socioeconomic status, defined by the median of the standardized household income distribution. HR = Hazard Ratio; CI = Confidence Interval. †*Salmonella* isolated from urinary tract or wound infections.
