# Supplementary material for: Increased colon cancer risk after severe Salmonella infection
Source: PLoS One. 2018 Jan 17;13(1):e0189721. doi: 10.1371/journal.pone.0189721 (PMC5771566; doi:10.1371/journal.pone.0189721)
Supplement: S9 Table — (DOCX) [file pone.0189721.s009.docx]

**S9 Table:** **Outputs from the univariate binomial regression analysis of pathology records.**

Results of the univariate binomial regression models predicting reported *Salmonella* infection among patients with colon cancer as a function of genetic and tumor pathological factors of the colon cancer patients using a three times larger gender- and age-matched colon cancer control group without reported *Salmonella* infection. Colon cancer cases per colon subsite affected with (Salm+) and without (Salm-) a reported Salmonella infection, risk ratio (RR) and 95% confidence interval (CI).

|  | **Colon cancer (overall)** | | | **Ascending & transverse colon** | | | **Descending & sigmoid colon** | | |
| --- | --- | --- | --- | --- | --- | --- | --- | --- | --- |
|  | **Salm+** | **Salm-** | **RR (95% CI)** | **Salm+** | **Salm-** | **RR (95% CI)** | **Salm+** | **Salm-** | **RR (95% CI)** |
| **IBD** |  |  |  |  |  |  |  |  |  |
| No | 60 | 190 | Reference | 44 | 141 | Reference | 16 | 49 | Reference |
| Yes | 5 | 4 | 2.43 (1.31-4.50)** | 4 | 3 | 2.54 (1.29-5.00)** | 1 | 1 | 2.16 (0.49-9.62) |
| **MSI** |  |  |  |  |  |  |  |  |  |
| No | 9 | 25 | Reference | 7 | 17 | Reference | 2 | 8 | Reference |
| Yes | 3 | 9 | 0.94 (0.30-2.92) | 3 | 9 | 0.86 (0.27-2.73) | 0 | 0 | ∞ |
| Unknown | 53 | 160 | 0.93 (0.49-1.77) | 38 | 118 | 0.83 (0.41-1.67) | 15 | 42 | 1.63 (0.24-11.03) |
| **Genetic predisposition** |  |  |  |  |  |  |  |  |  |
| No | 1 | 9 | Reference | 1 | 7 | Reference | 0 | 2 | Reference |
| Yes | 4 | 11 | 2.71 (0.35-20.94) | 4 | 5 | 3.61 (0.50-26.21) | 0 | 6 | ∞ |
| Unknown | 60 | 174 | 2.60 (0.40-17.04) | 43 | 132 | 1.96 (0.30-12.61) | 17 | 42 | ∞ |
| **Tumor stage** |  |  |  |  |  |  |  |  |  |
| 0-I | 15 | 23 | Reference | 12 | 17 | Reference | 3 | 6 | Reference |
| II | 12 | 54 | 0.44 (0.23-0.85)* | 11 | 43 | 0.47 (0.23-0.93)* | 1 | 11 | 0.25 (0.03-2.10) |
| III | 25 | 74 | 0.60 (0.35-1.01) | 15 | 51 | 0.51 (0.27-0.96)* | 10 | 23 | 0.96 (0.33-2.84) |
| IV | 11 | 42 | 0.49 (0.25-0.95)* | 9 | 32 | 0.50 (0.23-1.03) | 2 | 10 | 0.50 (0.10-50.83) |
| Unknown | 2 | 1 | 1.83 (0.69-4.89) | 1 | 1 | 1.31 (0.29-6.00) | 1 | 0 | 5.67 (0.63-50.83) |
| **Tumor differentiation** |  |  |  |  |  |  |  |  |  |
| Well-differentiated | 37 | 98 | Reference | 29 | 76 | Reference | 8 | 22 | Reference |
| Undifferentiated | 15 | 57 | 0.76 (0.45-1.29) | 13 | 46 | 0.79 (0.45-1.41) | 2 | 11 | 0.55 (0.13-2.32) |
| Unknown | 13 | 39 | 0.91 (0.52-1.58) | 6 | 22 | 0.77 (0.35-1.69) | 7 | 17 | 1.13 (0.46-2.77) |

§ Corrected only for the matching variables gender and age at cancer diagnosis. IBD = inflammatory bowel disease; tumor stage based on TNM classification); MSI = microsatellite instability; genetic predisposition refers to mutations in the Ras/Raf/Mapk pathway. *p-value <0.05; **p-value <0.01; ***p-value <0.001.
