# Supplementary material for: Increased colon cancer risk after severe Salmonella infection
Source: PLoS One. 2018 Jan 17;13(1):e0189721. doi: 10.1371/journal.pone.0189721 (PMC5771566; doi:10.1371/journal.pone.0189721)

**S1 Fig: Incidence of colon cancer according to age in the Dutch population between 1999 and 2016 (in bars) and projected standardized incidence ratio (SIR) and 95% confidence interval (CI) for colon cancer among those infected in the same decade of life with *Salmonella* (dots).**


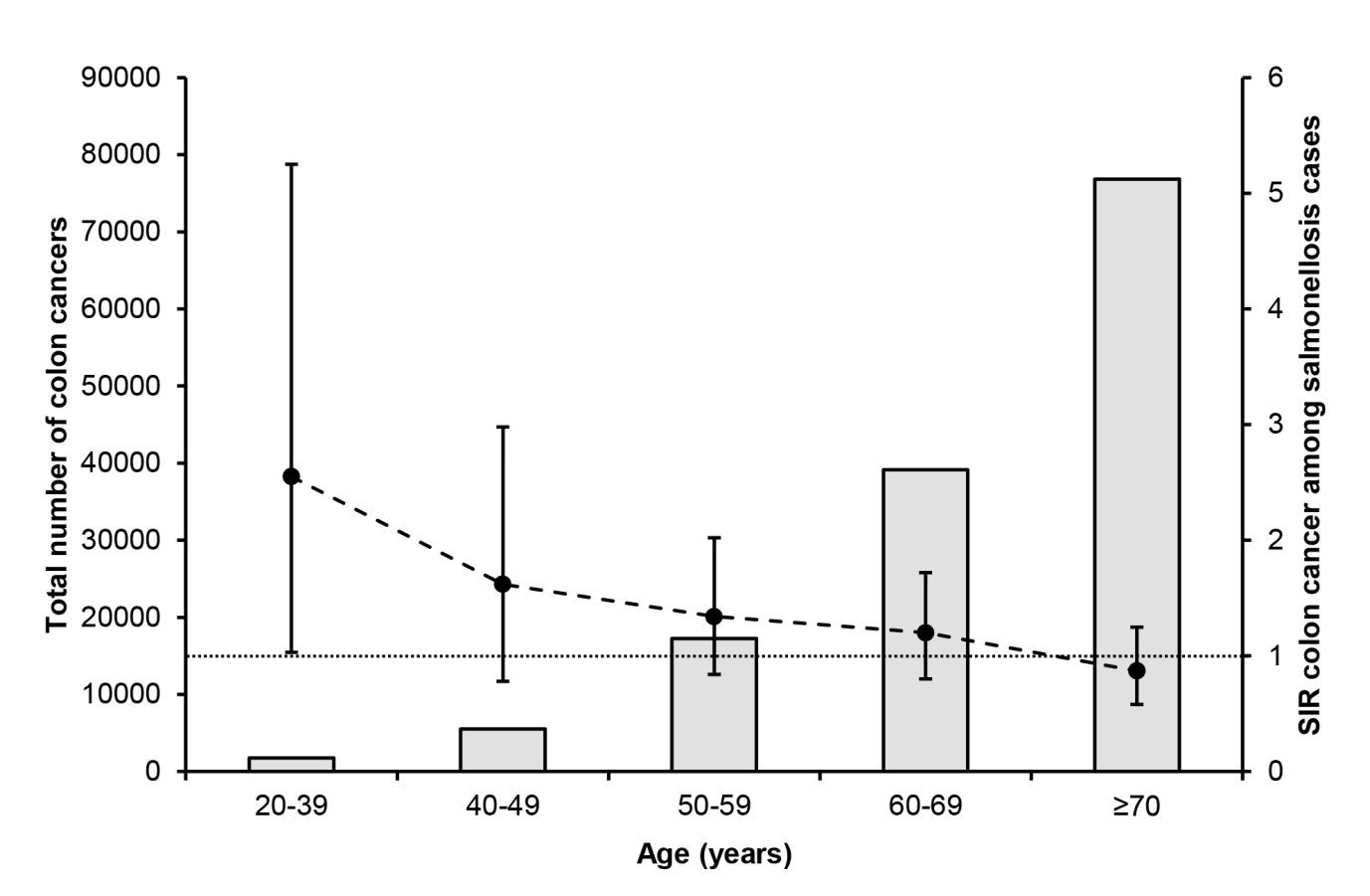

Supplement: S1 Fig — (DOCX) [file pone.0189721.s010.docx]
