# Supplementary material for: Increased colon cancer risk after severe Salmonella infection
Source: PLoS One. 2018 Jan 17;13(1):e0189721. doi: 10.1371/journal.pone.0189721 (PMC5771566; doi:10.1371/journal.pone.0189721)

**S2 Fig: Cumulative incidence of colon cancer by attained age in patients with reported *Salmonella* infection.**


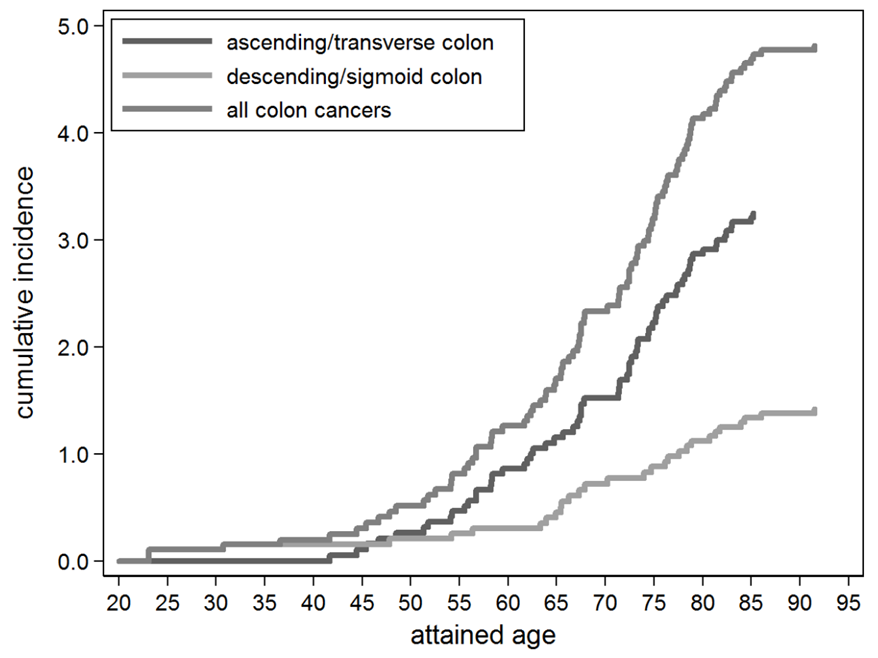

Supplement: S2 Fig — (DOCX) [file pone.0189721.s011.docx]
