# Supplementary material for: Increased colon cancer risk after severe Salmonella infection
Source: PLoS One. 2018 Jan 17;13(1):e0189721. doi: 10.1371/journal.pone.0189721 (PMC5771566; doi:10.1371/journal.pone.0189721)

**S3 Fig: Cumulative incidence of cancer in the descending and sigmoid parts of the colon**

Cumulative incidence of cancer in the descending/sigmoid colon over attained age in patients with a reported history of *Salmonella* infection and in the general population. Inset: cumulative incidence of cancer in the descending/sigmoid colon in patients with any *Salmonella* serovar infection and in the general population. Main graph: cumulative incidence of cancer in the descending/sigmoid colon in patients infected with the two major *Salmonella* serovars (Enteritidis and Typhimurium), the other less often diagnosed *Salmonella* serovars combined, and in the general population.


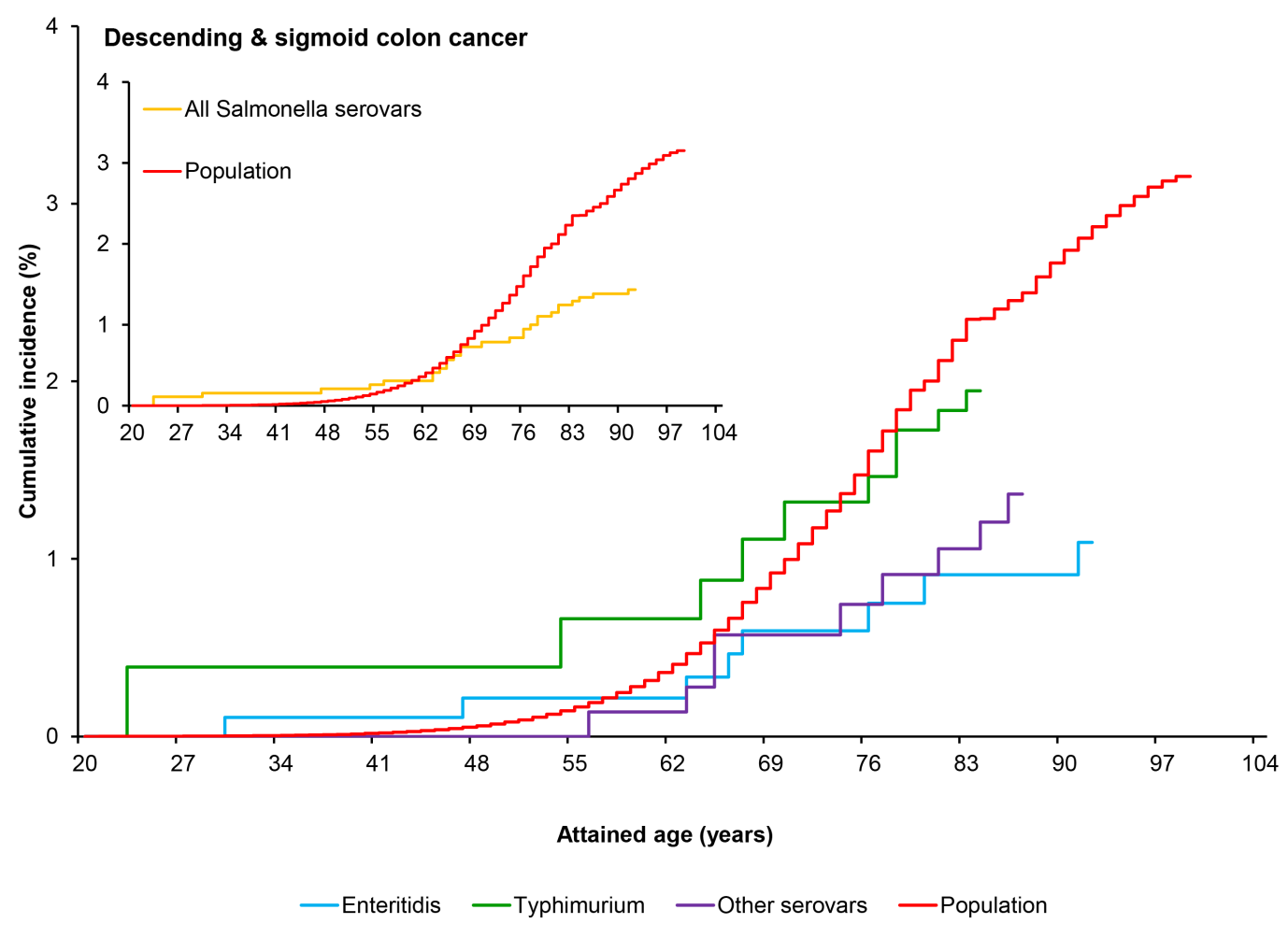

Supplement: S3 Fig — (DOCX) [file pone.0189721.s012.docx]
